# Environmental DNA-based biomonitoring of Cuban *Crocodylus* and their accompanying vertebrate fauna from Zapata Swamp, Cuba

**DOI:** 10.1038/s41598-023-47675-8

**Published:** 2023-11-22

**Authors:** Etiam Pérez-Fleitas, Yoamel Milián-García, Gustavo Sosa-Rodríguez, George Amato, Natalia Rossi, Matthew H. Shirley, Robert H. Hanner

**Affiliations:** 1 Enterprise for the Conservation of the Zapata Swamp, Ciénaga de Zapata, Matanzas Cuba; 2 Department of Integrative Biology, University of Guelph, Guelph, ON Canada; 3 Institute for Comparative Genomics, American Museum of Natural History, Central Park West at 79th Street, New York, NY 10024 USA; 4 Wildlife Conservation Society, 2300 Southern Blvd., Bronx, NY 10460 USA; 5 National Forensic Science Technology Center, Global Forensics and Justice Center, Florida International University, 8285 Bryan Dairy Rd #125, Largo, FL 33777 USA

**Keywords:** DNA sequencing, Next-generation sequencing

## Abstract

Crocodylians globally face considerable challenges, including population decline and extensive habitat modification. Close monitoring of crocodylian populations and their habitats is imperative for the timely detection of population trends, especially in response to management interventions. Here we use eDNA metabarcoding to identify the Critically Endangered *Crocodylus rhombifer* and the Vulnerable *C. acutus*, as well as vertebrate community diversity, in Cuba’s Zapata Swamp. We tested four different primer sets, including those used previously in *Crocodylus* population genetic and phylogenetic research, for their efficiency at detecting crocodylian eDNA. We detected *C. rhombifer* eDNA in 11 out of 15 sampled locations within its historical geographic distribution. We found that data analyses using the VertCOI primers and the mBRAVE bioinformatics pipeline were the most effective molecular marker and pipeline combination for identifying this species from environmental samples. We also identified 55 vertebrate species in environmental samples across the four bioinformatics pipelines— ~ 85% known to be present in the Zapata ecosystem. Among them were eight species previously undetected in the area and eight alien species, including known predators of hatchling crocodiles (e.g., *Clarias* sp.) and egg predators (e.g., *Mus musculus*). This study highlights eDNA metabarcoding as a powerful tool for crocodylian biomonitoring within fragile and diverse ecosystems, particularly where fast, non-invasive methods permit detection in economically important areas and will lead to a better understanding of complex human-crocodile interactions and evaluate habitat suitability for potential reintroductions or recovery programs for threatened crocodylian species.

## Introduction

Biodiversity conservation faces multiple challenges caused by anthropogenic habitat degradation, the reduction of natural land due to agricultural expansion, and the introduction of invasive alien species^1^. In addition to the human-driven causes, the lack of information on species distribution and habitat requirements limits our ability to mitigate conservation threats^2^. Reptiles are a significant component of biodiversity, and despite the global decline in their natural populations, our understanding of their conservation status is largely unknown^3^. Living crocodylians are among the most prominent apex predators and also among the most threatened groups of vertebrate taxa, with 26 currently recognized species—46% (12 out of 26) of them belonging to the genus *Crocodylus*^4,5^, including four Critically Endangered and two Vulnerable species^6^. All of the most threatened crocodylian species are targeted by dedicated conservation efforts, often including conservation breeding for reintroduction purposes, translocations, and increased law enforcement^7^. Among the species that are not considered threatened, most are managed as part of sustainable use programs and are harvested for their meat and skins in a considerable global trade^7^. As a result, integral population monitoring programs, that include crocodylians and their accompanying fauna (e.g., diversity of potential vertebrate prey), are critical for evaluating the success of intense conservation interventions or ensuring sustainable offtake and trade. Unfortunately, crocodylians are also highly cryptic and, especially for the threatened species, are often very difficult to detect and/or occupy remote, difficult-to-access habitats^7^, which can impede monitoring and research efforts. While standardized crocodylian survey methods have been refined through years of work (e.g., aerial diurnal surveys and nocturnal spotlight surveys^7^), more rapid, cost-efficient, and non-invasive survey methods may be more effective ways to monitor threatened crocodylian species.

Among crocodylians, the Cuban crocodile (*Crocodylus rhombifer*) is a Critically Endangered species^8^ currently endemic to Cuba, where it is only found in the freshwater habitats of Zapata Swamp and Isla de la Juventud^9^. The latter population results from a reintroduction program started in 1990 in an area where the species was previously extirpated^9^, making Zapata Swamp’s population the last natural remanent. The geographic distribution of Cuban crocodiles has been drastically reduced since the Pleistocene when, according to fossil records, they could be found in the central region of Cuba^10^ and other Caribbean islands, such as The Bahamas^11^. The Cuban crocodile shares its range in the Zapata Swamp, the largest insular Caribbean wetland, with the American crocodile (*Crocodylus acutus*). This species is broadly distributed around the Cuban archipelago and throughout the Caribbean^12,13^. These two species are well known to hybridize in the wild and captivity^14^. Hybrid identification remains a challenge that can only be disentangled using molecular approaches^14^. Despite the widespread hybridization observed between populations of the Cuban and American crocodile in the wild and in captivity, there is substantial empirical evidence to distinguish non-hybrid populations of these species on the basis of ecological, morphological, and ethological factors^9,15^.

Habitat modification constitutes one of the main threats to all crocodylians occurring in Cuba^16^. The construction of artificial water channels, saline intrusion, contamination by agrochemicals, and the introduction of exotic species are some of the main modifications negatively impacting the Cuban crocodile population in Zapata Swamp^9^. These modifications have resulted in increased saltwater incursion and wider distribution by *C. acutus* in Zapata Swamp, which are reducing the occurrence of *C. rhombifer* and increasing the frequency of hybridization^14^. In spite of this, our understanding of the actual distribution of *C. rhombifer* in the Zapata Swamp dates to studies now three decades old^9^. Monitoring Cuban crocodiles in Zapata Swamp is complicated by logistics, an exceptionally challenging habitat to access and navigate, and the rarity of the species, requiring extended periods in the field for even minimal detections. Determining an efficient monitoring approach capitalizing on occupancy within sectors of Zapata Swamp will enable the modelling of past and future trends to achieve more efficient conservations and interventions in favor of this Critically Endangered species.

In the last decade, environmental DNA (eDNA) has become widely used to study species occurrence patterns, especially in aquatic ecosystems^17^. eDNA metabarcoding is a non-invasive method of monitoring biodiversity, making its application increasingly practical and attractive for surveying endangered species and fragile ecosystems^18,19^. Also, High-Throughput Sequencing (HTS) advances are considerably reducing the costs of applying eDNA metabarcoding methods^19^. Further, its application overcomes many limitations of traditional methods, including the need for morphology-based identifications requiring trained taxonomists and considerable time^2,20–22^. However, as it is a method relying on degraded DNA, low detection rates can limit its use, and detection varies among species, habitats, and specific methods^23^. Nevertheless, eDNA has been effectively used to detect the presence of fish^24,25^, amphibians^26^, turtles^27^, and snakes^28^, many of which are crocodylian prey. While eDNA metabarcoding has been broadly applied to fish and other taxa, application to herpetofauna is less common^29,30^, partly because commonly used primers seem to miss them and therefore studies more commonly use targeted assays. A recent study illustrated through a controlled environment study that crocodiles shed enough DNA to be detected in water samples, that the DNA is stable for several days even under high UV index, and that eDNA-based methods allow for discrimination among crocodylian species—highlighting its potential utility for wild crocodylian populations^31^.

In the present study, we investigated the utility of eDNA as a detection and monitoring tool for threatened crocodylians and their prey, using the Cuban crocodiles of Zapata Swamp as a test case. To do this, we tested the ability of established crocodylian mitochondrial DNA (mtDNA) primers to amplify *C. rhombifer* and *C. acutus* DNA in environmental samples. We compared the results when using croc-specific and versatile primers for detecting crocodylian through an eDNA metabarcoding approach applied to the same environmental samples*.* The versatile primer sets have not yet been used to detect crocodylians or distinguish between syntopically occurring and closely related crocodylian species from complex environmental samples; thus, proof of concept is warranted. Among the primers, we used standard vertebrate primers to describe the full vertebrate diversity detectable through metabarcoding at the sampling sites to potentially monitor crocodylian prey and other species interactions. This latter step, in particular, can help inform future reintroduction efforts through an objective site suitability assessment.

## Results

### Cuban crocodile detection based on eDNA metabarcoding

The Croc_COI and Croc_CR analyses generated 18,574,810 and 14,971,796 sequences, while 50,061,458 and 38,389,688 sequences were obtained for VertCOI and 12S, respectively, considering all analyzed biological and technical replicates and controls. Not quantifiable DNA was found in the control extracts; however, spurious amplifications were detected. All primer sets amplified eDNA fragments extracted from the water samples at the expected fragment sizes.

Analyzing the Croc_COI primer set in the mBRAVE pipeline resulted in only a single assignment (450 bp, 100% similarity) to *C. rhombifer* (BOLD: ACI0039). This sequence came from a single technical replicate from one of the biological replicates taken from the positive control pool at the ZSCF. None of the sequences generated from any samples taken from any of the 15 surveyed localities in Zapata Swamp resulted in BIN assignment to either *C. rhombifer* or *C. acutus* using the mBRAVE pipeline. Analyzing Croc_COI and Croc_CR amplified sequences within the Geneious platform resulted in matches to four (two for each mitochondrial marker) of the 81 reference sequences to the target crocodile species (Supplementary Information 1). Only Croc_COI amplified eDNA sequences from Laguna de Vitorino and from the ZSCF positive control pond resulted in positive matches to a *C. acutus* (GenBank accession number HQ595043.1) and *C. rhombifer* × *C. acutus* hybrid—morphology-based classification—(GenBank accession number HQ595047.1) reference haplotypes. Additionally, sequences generated from Laguna de Vitorino and ZSCF positive control pond based on Croc_CR matched two reference *C. rhombifer* haplotypes (GenBank accession numbers EU499910.1 and JF315339.1).

The VertCOI primer successfully amplified *C. rhombifer* eDNA fragments in water samples collected in the Zapata Swamp (Table 1), including in at least one technical replicate and one of the two biological samples from 11 out of the 15 sampling locations (Table 1). Laguna de Vitorino and Zanja del Diez were the only sites where eDNA fragments of *C. rhombifer* were amplified from both biological sample replicates and all six technical PCR replicates. *Crocodylus acutus* was not detected in any eDNA samples using the VertCOI primers.

**Table 1:** Representation of *C. rhombifer* eDNA sequence detection at each surveyed locality in each technical and biological replicate sampled when using VertCOI primers and after data analysis in mBRAVE. The Table did not include localities where no *C. rhombifer* eDNA detection was found in any replicate.

Locality	Tech replicate	1st biological replicate			2nd biological replicate		
		Reads	ML	MS (%)	Reads	ML	MS (%)
Zanja del Diez	I	16,060	184.95	99.89	9422	184.96	99.81
	II	1	185	100	22,759	184.95	99.89
	III	2	185	100	3	185	100
Zanja del Nueve	I	–	–	–	1	185	100
	II	1	185	100	4	185	99.86
	III	–	–	–	2	185	100
Estamento	I	–	–	–	–	–	–
	II	–	–	–	–	–	–
	III	–	–	–	2	184.5	100
Punta Arena	I	–	–	–	–	–	–
	II	–	–	–	–	–	–
	III	–	–	–	1	185	100
Estero de Punta Arena	I	2	185	100	–	–	–
	II	–	–	–	–	–	–
	III	2	185	100	–	–	–
Laguna de Vitorino	I	35,841	184.96	99.91	66,164	184.96	99.93
	II	25,258	184.95	99.91	48,288	184.96	99.93
	III	37,499	184.95	99.92	91,116	184.95	99.93
Lagunitas	I	4	185.25	99.73	–	–	–
	II	10	185	99.89	–	–	–
	III	13	185	100	–	–	–
Zanja de Santo Tomás	I	1	185	100	–	–	–
	II	3	185	100	–	–	–
	III	–	–	–	–	–	–
Canal de los Patos I	I	12	184.92	99.82	5	185	99.89
	II	–	–	–	6	185	100
	III	–	–	–	5	185	99.78
Canal de los Patos II	I	5	185	99.46	–	–	–
	II	2	185	99.46	2	185	100
	III	6	185	99.91	1	185	100
Laguna Nueva	I	–	–	–	10	185	99.89
	II	–	–	–	31	184.77	99.86
	III	–	–	–	20	185	100

Average sequence length (ML), average mean similarity (MS%) to the reference haplotypes, and the total number of reads (Reads) generated from each technical replicate are included.

### Habitat biodiversity

**Figure 1:**
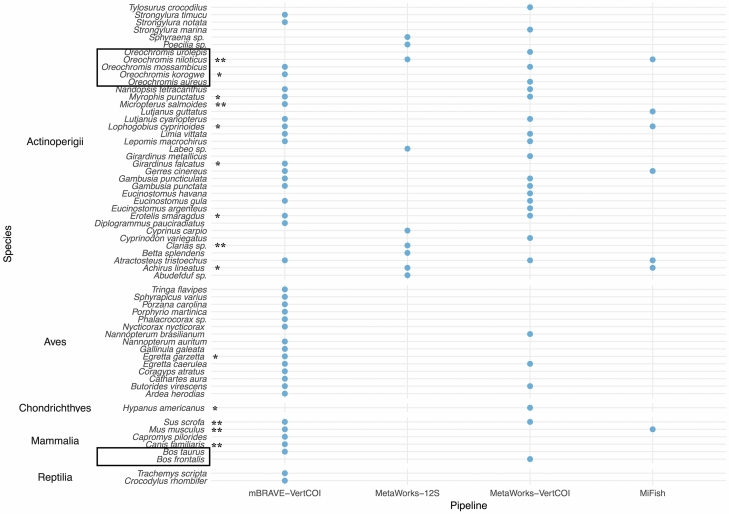
List of identified taxa from the eDNA fragments amplified from the water samples collected in this study after using two mitochondrial regions (COI and 12S) and three bioinformatic pipelines for data analysis. The taxa within the boxes represent species for which the markers used resulted in lower species resolution. *Newly recorded species, **Invasive taxa.

In total, we detected 55 species from 43 genera, 29 families, 25 orders, and 5 classes from eDNA fragments found in the sampled aquatic ecosystems of Zapata Swamp (Fig. 1). These included 47 (62.7%) of the 75 species known to inhabit the aquatic ecosystems of Zapata Swamp, as well as eight newly recorded species (Fig. 1).

Using the VertCOI primers and the mBRAVE pipeline, we identified four classes, 20 orders, 24 families, 35 genera, and 39 species. Sites where eDNA fragments of *C. rhombifer* were amplified showed slightly lower average richness values within each taxonomic category compared to sites where *C. rhombifer* was not identified; however, these differences were not statistically significant (class: W = 23.5, p = 0.88; order: F = 1.82, Critical F = 8.78, p = 0.33; family: F = 2.72, Critical F = 8.78, p = 0.27; genus: F = 1.49, Critical F = 8.78, p = 0.41; species: F = 1.97, Critical F = 8.78, p = 0.31). Using the VertCOI primers and the MetaWorks pipeline, we identified 26 species distributed in 22 genera, 16 families, 13 orders, and 5 classes. Again, diversity was not significantly different for any taxonomic category between sites with and without *C. rhombifer* (class: W = 17.5, p = 0.7594; order: F = 0.46, Critical F = 0.26, p = 0.16; family: F = 0.56, Critical F = 0.26, p = 0.22; genus: F = 0.57, Critical F = 0.26, p = 0.22; species: F = 0.70, Critical F = 0.26, p = 0.29).

The MiFish pipeline identified seven species (with high or moderate confidence; ≥ 97% identity) belonging to seven genera, seven families, seven orders, and two classes across all surveyed localities. We analyzed the same datasets on the MetaWorks pipeline and identified four species [with high bootstrap support (≥ 0.97)] belonging to four genera, four families, four orders, and one class from amplified eDNA fragments at the surveyed localities. We also identified six fish genera (*Clarias*, *Labeo*, *Poecilia*, *Eleotri*, *Sphyraena*, and *Abudefduf*) and three families (Clariidae, Sphyraenidae, and Pomacentridae), but were unable to assign them to specific species.

## Discussion

Environmental (e)DNA has become a standard non-invasive technique for rapidly surveying single-species or multispecies communities around the world^19,32^. However, only one study published to date has assessed the potential for monitoring crocodylians using eDNA^31^. The mentioned study used species-specific qPCR assays to amplify crocodylian eDNA fragments from water samples collected in a controlled environment. Here, we show for the first time the potential of an eDNA metabarcoding approach to rapidly detect Cuban crocodiles and describe species richness (i.e., potential prey) found in their habitats. Our results provide both proof of concept that eDNA in natural settings detects crocodylians (in our case, mainly Cuban crocodiles), and that well-established metabarcoding assays and bioinformatic pipelines could be an immediately accessible solution.

Among the primer sets and bioinformatic pipelines we evaluated for the detection and identification of the threatened crocodylians of Cuba, the VertCOI primers analyzed in mBRAVE performed the best. The VertCOI primer cocktail amplifies a ± 185 bp sequence within the standard COI barcode region, which has several advantages. First, eDNA is a degraded DNA approach, meaning that shorter fragments are more likely to successfully amplify^19^. Second, as the mitochondrial COI region (5ʹ) has been the standard animal barcoding marker for over 20 years^33,34^, a substantial reference database facilitates species identification in the Barcode of Life Data System (BOLD^35^). Using these primers and analytical pipeline, we successfully amplified eDNA sequences from 11 of 15 sites and identified them as *C. rhombifer*. However, none of the amplified *C. rhombifer* sequences could be matched to previously identified COI haplotypes^36,37^. This is easily explained by the lack of overlap between the fragment amplified by VertCOI in the BOLD database, which targets the 5ʹ COI region^35^, compared to that amplified by Croc_COI, which targets the 3ʹ COI region^36,37^. These previously reported haplotype-specific sequences are also not present in the mBRAVE reference datasets. We also failed to amplify sequences that could be assigned to *C. acutus* despite its known presence in at least seven of our 15 sampled sites^15,16^. This is most likely because the only 5ʹ COI haplotypes in the BOLD reference database represent continental *C. acutus*, which are known as highly divergent from the Antillean evolutionary lineage^14,38,39^. It also highlights the need for DNA reference database completeness, particularly representing *Crocodylus* haplotype diversity to enhance species identifications based on the target molecular region.

This lack of specific reference sequences is a limiting factor for the VertCOI primers, at least for New World crocodylians. While this issue may be resolved using species-specific primers and customized reference databases, the existing Croc_COI and Croc_CR failed almost entirely to amplify crocodylian eDNA amongst our samples. Confirmation of their ability to amplify crocodylian DNA in environmental samples would have enabled future researchers to conduct eDNA metabarcoding in the range of virtually any crocodylian species globally without the need to develop species-specific primers or assays. However, their lack of success is likely because the target fragment sizes are too long, which results in unmerged end reads, lack of primers expected specificity when used in environmental samples or, more likely, with fragments too small for the primers to bond due to the degraded state of the DNA in the environment. As a result, either novel species-specific primers targeting smaller fragments and/or species-specific qPCR/dPCR assays may be more successful. Either way, a higher representation of *Crocodylus* haplotypes in standard DNA reference databases, such as that utilized by mBRAVE, will be needed.

Crocodiles spend most of their time in the water, only going ashore to bask, find new water reservoirs during the dry season, capture prey near the shore, or nest^7^. Additionally, courtship, mating, seasonal movements, resting, and feeding behaviors are carried out in the water, specifically on the surface^15^. Crocodiles also move between sites by swimming on the water’s surface^9^. During these behaviors, a significant amount of eDNA might be expected to be shed in the water column, which could increase the probability of finding eDNA fragments at the top of the water column and explains why the samples were collected on the water’s surface of the crocodiles’ ecosystem contrary to sample in depth. However, further research will be needed to evaluate whether crocodylians’ shed eDNA rapidly settles and, contrary to our assumption, is more likely to be found at greater depths.

One of the main limitations of using eDNA to detect threatened crocodylians is that low population abundance leads to low DNA concentrations in the environment^40^. In these cases, the probability of detecting rare DNA can be augmented by increasing the number of samples collected and the number of PCR replicates and/or cycles^41,42^. Although targeted detection sensitivity and specificity could also be boosted by alternative molecular techniques such as digital PCR^43^, it lacks the capacity for multispecies detection (e.g., *Crocodylus* and their diet) and was considered out of the scope of the present study. Other factors, such as DNA degradation^44^ and low DNA concentration caused by flowing water, can also lead to misrepresentation of species occurrence in surveyed ecosystems^20,45,46^. During sampling, for example, four crocodiles (± 1.5 m total length) were observed in Estamento, presumably all *C. rhombifer*. In spite of this visual confirmation, only two eDNA fragments were recovered in one biological replicate at this site. The Estamento ponds are considered shallow and have very little vegetation cover compared to the other sampled sites, suggesting that higher temperatures and increased UV exposure may result in increased eDNA degradation. Three other sampled areas (Majá Parado, Zona de Liberación, and Canal) are lotic ecosystems where no eDNA fragments were amplified. These sites are located within a canal system through which a large volume of water is carried from the eastern to the western region of Zapata Swamp. The movement of water, in this case, may simply over disperse and dilute the already rare crocodylian eDNA, decreasing its probability of being sampled. Future eDNA studies of threatened crocodylians should consider increasing sampling efforts (multiple points per site), sampling from different depths, and collecting larger volumes of water to overcome these issues.

In spite of these limitations, we detected *C. rhombifer* at 11 of our 15 sampled localities, including Estamento, Punta Arena, and Estero de Punta Arena. These latter sites are reported as low-density areas of crocodiles within the geographic range^9^, highlighting the utility of the current approach to detecting Cuban crocodiles at sites with low density and low observation probability using traditional methods like nocturnal spotlight surveys. Despite the historical reports of the presence of crocodiles in all the sampled localities, four of them had no detections of *Crocodylus* eDNA fragments. These four sites correspond to those with the lowest density of crocodiles traditionally observed^9^, but also the higher anthropogenic pressures such as illegal hunting and habitat modification due to economic activities. Consequently, it is as yet unclear if crocodiles remain at these sites. In either case, our eDNA metabarcoding surveys are providing needed, updated information on the distribution of threatened Cuban *Crocodylus* in this critical ecosystem after nearly 30 years of no population updates.

### Biodiversity within the historical distribution areas of the genus *Crocodylus* at Zapata Swamp

A secondary advantage of using eDNA metabarcoding to survey rare and threatened species dependent on conservation intervention is the concurrent ability to rapidly assess biological communities, including the presence and diversity of key resources (e.g., prey or habitat) needed by these species^2,22,44,46^ or the presence of potentially injurious or competing species, including invasives^28,47,48^. To date, the biodiversity of the ecosystems within the Zapata Swamp has been explored using only traditional methods (e.g., Targarona 2013), limiting the chances of detecting slight shifts in the biological community structure and cryptic or rare species. Through eDNA, we were able to update our understanding of Zapata’s biodiversity, including through the detection of eight as yet reported species in the sampled sites, such as goldbelly topminnow (*Girardinus falcatus*). In total we detected 55 species, 16 more than the highest number identified using a single bioinformatics platform (Fig. 1). Our use of multiple primer sets and bioinformatic pipelines allowed us to overcome limitations inherent in any one reference database. For example, the MetaWorks trained datasets do not contain any *C. rhombifer* or *C. acutus* reference sequences, thus necessitating additional databases for full and accurate species identification.

The 55 detected species are all potential prey for Cuban *Crocodylus*^9,49^. A better understanding of the distribution of important prey, especially for *C. rhombifer*, could facilitate decision-making for reintroduction program success. We know already that our results are likely to be painting an incomplete picture of prey availability. First, our exclusively aquatic sampling regime may limit our ability to detect terrestrial species, also known as part of the *C. rhombifer* diet^15,16^. Second, the primer sets/cocktails used for COI DNA amplification here are not versatile enough to amplify mollusk and amphibian DNA effectively, despite their observed presence in the sampled sites. Additional work is needed, which may also aid in the search for elusive endemics, such as the Critically Endangered dwarf hutia (*Mesocapromys nanus*) and Zapata Rail (*Cyanolimnas cerverai*). eDNA metabarcoding has allowed us to confirm the presence of three species within the genus *Eucinostomus* (*Eucinostomus argenteus, Eucinostomus gula,* and *Eucinostomus havana*), which are not reported beyond the genus level in Zapata Swamp regular species inventories, due difficult identification using traditional morphological methods. Altogether, our study reinforces the utility of eDNA-based biomonitoring in ecosystems across the Cuban archipelago.

Exotic and/or invasive species are increasingly problematic for conservation management, especially where they have a high potential to negatively impact native species^50^, and alien species are among the most critical threats to reptile conservation in many regions^3^. Our eDNA results detected at least three species that have never been observed or detected in Cuba to date, much less the Zapata Swamp area, including little egret (*Egretta garzetta*) and korogwe tilapia (*Oreochromis korogwe*). These would be considerable extralimital observations for these species, including their known extralimital range, and thus, assignment to them at the species level may be because of issues with the reference databases or lower marker resolution to distinguish species within these genera. The other species are the red-eared slider (*Trachemys scripta*) and fish of the genus *Clarias* (from 10 of the 15 sampled localities). The latter is a known invader in Cuba^51^, and this genus is a known predator of hatchling crocodiles, in addition to competing with young crocodiles for food resources (personal observations). In the case of the former, our approach did not allow us to distinguish the confidence of species assignment—a known limitation with eDNA studies that must refer to BOLD and GenBank reference sequences, among others. In the case of closely related species where one or more are absent from reference databases, sequences can be assigned to erroneous species or to more than one species, reducing confidence in reporting biodiversity accurately. For example, *Trachemys scripta* has been reported as introduced in the Cayman Islands and Puerto Rico^52^, but never in Cuba. Though 2958 eDNA fragments amplified using VertCOI primers had 99.11% mean similarity with the reference sequence BOLD:AAK2050; this sequence has been variably assigned to four different *Trachemys* species from the Caribbean region, including the endemic *T. decussata*. It is unclear at this time if our results signal a new species introduction or, most likely, if only the 185 bp COI molecular fragment lacks enough resolution to distinguish closely related *Trachemys* species unambiguously.

## Conclusions for conservation of Cuban *Crocodylus*

Understanding how the environmental variables affect the use and selection of the habitat by crocodiles is of particular interest for improved management of the genus *Crocodylus* in Cuba. In the case of the Critically Endangered *C. rhombifer,* it permits the identification of appropriate locations for recovery or reintroduction initiatives. The benefits of eDNA metabarcoding are significant in both large spatial-scale studies and in fragile ecosystems. For instance, we were able to rapidly detect crocodiles at sites that are difficult to access and would normally require days of searching due to historically low abundance. Our inability to detect crocodiles at other sites may be due to true absences or may be due to false negatives for species resulting from partial PCR inhibition or DNA degradation due to environmental factors^2,40^. We also have shown that eDNA metabarcoding allows for quick identification of invasive species to inform appropriate management and mitigation decisions. A better understanding of invasive species distribution in Zapata Swamp will facilitate focusing conservation efforts and limited resources on the most sensitive areas, including *C. rhombifer* breeding sites.

The application of multi-marker approaches for eDNA metabarcoding, such as the one conducted in the present study, allowed for the detection of *Crocodylus*, as well as a comprehensive list of aquatic prey and invasive species in their habitat. Moving forward, *Crocodylus* molecular biomonitoring programs will benefit from building custom COI reference databases. In the future, it may also be possible to increase the target eDNA fragment length within the standard animal barcode region to better reconstruct specific haplotypes using second-generation HTS technology. Unfortunately, hybrid identification will not be possible using standard mitochondrial fragments. It would eventually require nuclear marker combinations, which are still undescribed in this system^39^ and may ultimately not be feasible using an eDNA approach. Altogether, the eDNA metabarcoding approach has proven helpful for detecting crocodiles in the wild, with a limited Cuban *C. acutus* detection rate due to the incomplete DNA reference databases. The multispecies identification approach has also allowed for identifying crocodiles’ prey and invaders impacting the natural ecosystems they inhabit. As a result, this approach facilitates a more rapid update of the geographic range of Cuban *Crocodylus* and for focusing conservation management efforts on more sensitive crocodile populations or suitable sites for recovery or reintroduction programs.

## Materials and methods

### Study area

**Figure 2:**
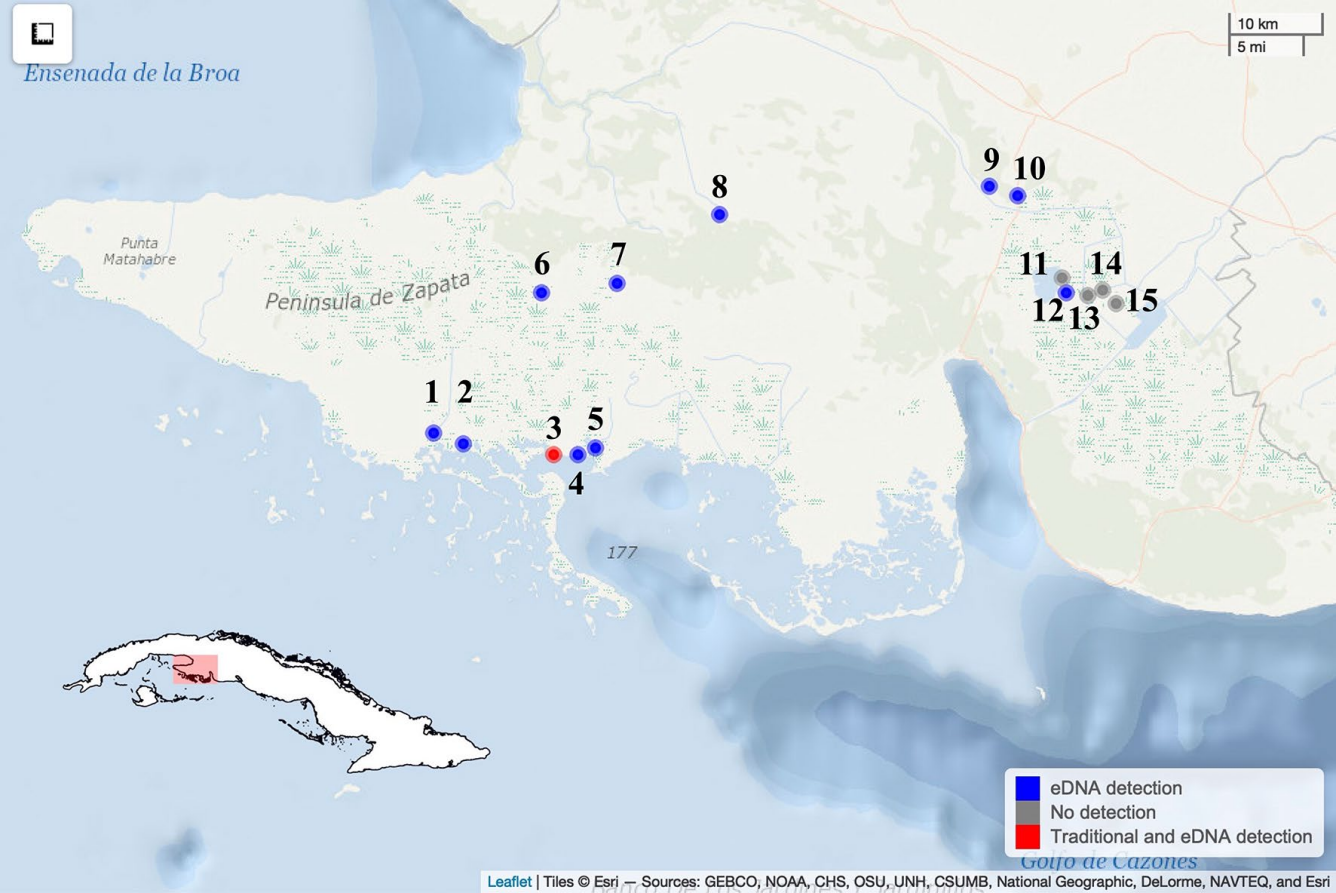
Sampled localities within the historical range of *Crocodylus rhombifer* at Zapata Swamp, Cuba. Blue dots indicate sites where crocodile eDNA was detected, grey dots represent sites without detection, and red dots illustrate the only site where crocodile eDNA and direct observation were possible to confirm crocodile presence. 1. Zanja del Diez, 2. Zanja del Nueve, 3. Estamento, 4. Punta Arena, 5. Estero de Punta Arena, 6. Laguna de Vitorino, 7. Lagunitas, 8. Zanja de Santo Tomás, 9. and 10. Canal de los Patos 2 and 1, 11. Laguna del Tesoro, 12. Laguna Nueva, 13. Canal in the “Canales de Hanábana” Faunal Refuge, 14. Majá Parado in the “Canales de Hanábana” Faunal Refuge, and 15. Zona de Liberación in the “Canales de Hanábana” Faunal Refuge.

**Figure 3:**
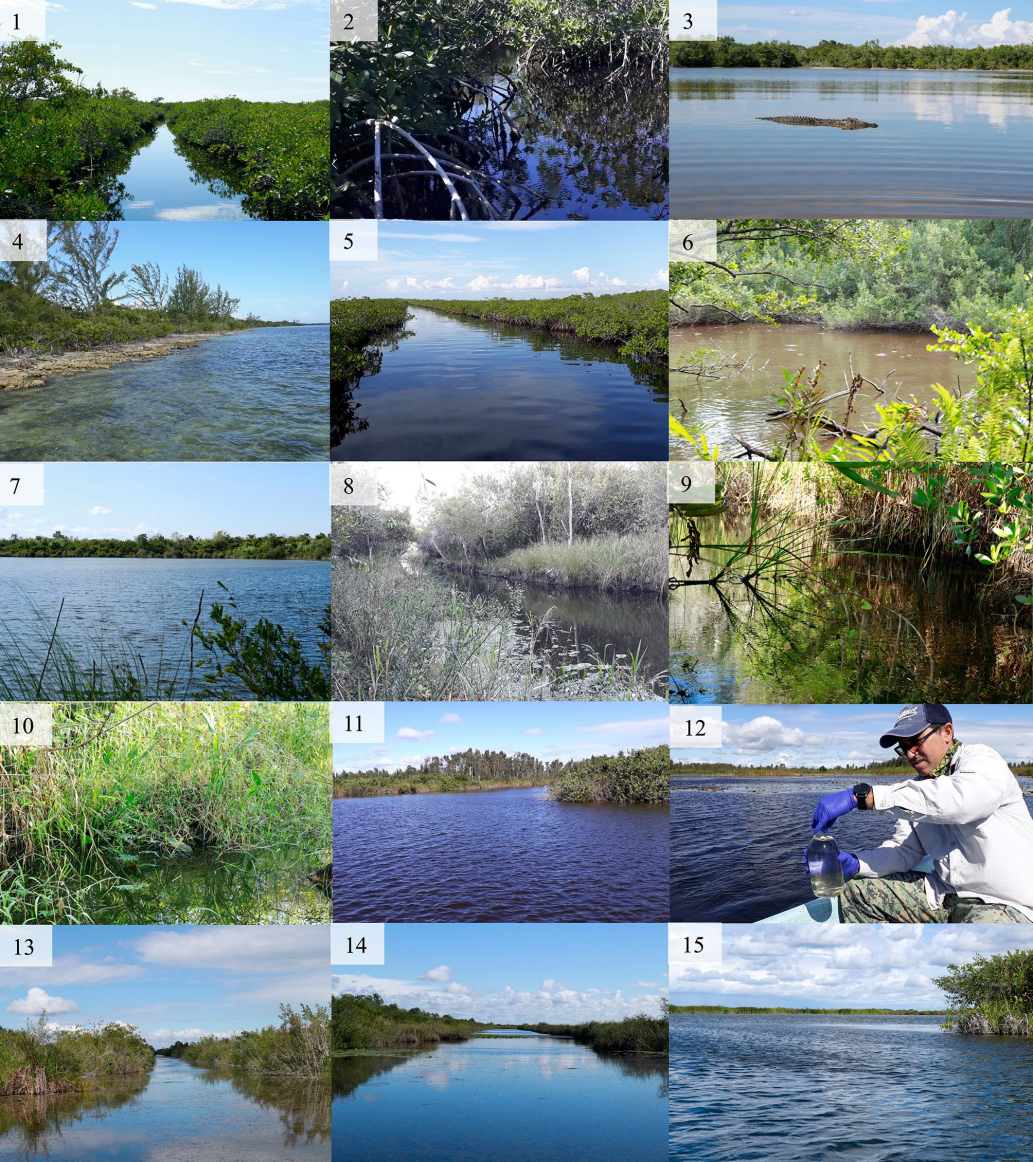
Representation of the wide range of field sites sampled in the present study. Panels are organized in the order of their description in the “Methods” section. From top left to right: 1. Zanja del Diez, 2. Zanja del Nueve, 3. Estamento, 4. Punta Arena, 5. Estero de Punta Arena, 6. Laguna de Vitorino, 7. Lagunitas, 8. Zanja de Santo Tomás, 9 and 10 Canal de los Patos 1 y 2, 11. Laguna del Tesoro, 12. Laguna Nueva, 13. Canal in the “Canales de Hanábana” Faunal Refuge, 14. Majá Parado in the “Canales de Hanábana” Faunal Refuge, and 15. Zona de Liberación in the “Canales de Hanábana” Faunal Refuge.

The Zapata Swamp, located in the southern region of Matanzas province, Cuba (Fig. 2), is the largest wetland in the insular Caribbean^53^. This area hosts high levels of biodiversity and endemism in several ecosystems, including freshwater lagoons and channels, grasslands, brackish water zones, estuaries, forests, and coral reefs^54^. Two hydro-climatic seasons regulate the water levels and the periods of flooding of these habitats that cover 4250 km^2^. We selected 15 sampling localities to collect water samples within the historical distribution of the genus *Crocodylus* at Zapata Swamp (Figs. 2, 3). A brief description of those areas is offered below:

#### Zanja del Diez

Canal (~ 9 km length) located in the southwestern region of the Zapata Swamp. Its average width is 2 m, and its depth ranges between 0.3 and 1 m. Salinity values depend on the distance from the sea, the tides, and the hydro-climatic season. This channel is considered the main genetic exchange route between crocodile populations of the inland freshwater ecosystems (typically *C. rhombifer*), and the coastal populations (typically *C. acutus*), so hybrids between Cuban *Crocodylus* are also expected^15^.

#### Zanja del Nueve

This canal differs from the rest by its construction on limestone rock following the terrain’s depressions and being narrower (~ 1 m). Grasslands of swamps and mangroves dominate this channel. Salinity values are subject to the same factors as in the Zanja del Diez. Mainly *C. rhombifer* and hybrids are expected in the site^15^.

#### Estamento

Calcic savannah with small lagoons with variable water levels and salinity associated with rainfall. The vegetation consists primarily of xeromorphic coastal and mangrove forests around the lagoons. Both Cuban *Crocodylus* and their hybrids are expected to be present^15^.

#### Punta Arena

Coastal strip with xeromorphic vegetation and sand spots without plant cover. It is considered an important crocodile nesting site; however, few American crocodile and hybrid individuals are observed by traditional methods (e.g., spotlight surveys) in this area^15,16^.

#### Estero de Punta Arena

System of canals through which the water of the northern region of Zapata Swamp drains towards the sea. The margins are covered with mangrove forests. Salinity values depend on the season of the year and the tides. Mainly crocodile hybrids are observed in this site^15,16^.

#### Laguna de Vitorino

Shallow pond with a surface area of 0.9 km^2^ covered mostly with mangroves and swamp forests. This lagoon is located within a savannah ecosystem, consisting of the only water reservoir during the dry period. American crocodiles and hybrids are typically observed in this site^15,16^.

#### Lagunitas

Small freshwater pond of approximately 0.03 km^2^ surrounded by swamp forest that floods seasonally, allowing the aquatic fauna to move to other ecosystems within the Zapata Swamp. Hybrid crocodiles are mainly expected in this area^15,16^.

#### Zanja Santo Tomás

Canal (12.8 km length) built in the early 1900’s to transport wood and charcoal. This channel crosses several ecosystems of swamp grassland and mangrove forests. Crocodile hybrids are frequently observed in this area^15^.

#### Canal de los Patos

Canal system built in the 1960s to facilitate the water runoff from the eastern swamp to the western swamp. Grasslands and swamp thickets dominate this area, with characteristic savannah vegetation, evergreen forests, and occasional mangroves. This locality is a frequent fishing spot, and there are reports of a few Cuban crocodile individuals, as well as hybrids^15,16^.

#### Laguna del Tesoro

One of Cuba’s most important natural lakes, with a surface area of 9 km^2^ and a depth of 10 m. Swamp grasslands surround it, showing a high degree of human impact due to tourism and fishing. It represents a typical habitat for the Cuban crocodile, although hybrids have also been found^15,16^.

#### Laguna Nueva

Pond spanning approximately 0.4 km^2^, and with a maximum depth of 4 m, connected to Laguna del Tesoro and Canales de Hanábana throughout channels. The fauna and flora composition are similar to that of Laguna del Tesoro, including Cuban crocodiles and hybrids^15,16^.

#### Fauna refuge “Canales de Hanábana”

Canal systems where Swamp grasslands cover 87% of the total area, and swamp forests and freshwater vegetation occupy the rest of the ecosystem. Currently, there are 175 species of terrestrial vertebrates reported in the area, 34 of which are endemic^54^. Our study includes three localities within this area: Majá Parado, Zona de Liberación, and Canal. Cuban crocodiles are mainly expected in this area, which have mainly been released as part of a reintroduction program (Figs. 2, 3).

### Sample collection, filtration, and storage

We collected 2 L of water from the surface from a single point in each locality selected randomly (Fig. 3) using two sterilized 1 L glass bottles, each of which was processed as a separate biological replicate. We also collected two samples of water (1 L each) from one pond (~ 9 m^3^) where 11 captive *C. rhombifer* (~ 2 m long) are kept at the Zapata Swamp Crocodile Farm (ZSCF), representing a positive control. Prior to sampling, we cleaned containers and lids with 50% bleach, rinsed 3× with distilled water, and sterilized them in an autoclave (121 °C and 1 atm) for 30 min. We fully immersed the containers in the water and put the lid right after it filled up wearing single-use gloves to collect the sample and avoid cross-contamination. We transported samples at environmental temperature in a box to limit UV exposure to a field lab for further processing. At the field lab, sampling personnel changed into clean clothing to prepare the work area.

We filtered samples using a vacuum pump and magnetic filtration cups with a three-piece manifold connected to a receiver container and a nitrocellulose mixed ester membrane filter (diameter 47 mm, pore size 1 μm) mounted between the mating surfaces of the filtration cups. We filtered each 1 L bottle per sampling locality separately, and the number of filters per 1 L sample differed due to the presence of suspended particulates. Filtering resulted in 83 total membranes, including the field negatives (2 L of distilled water filtered for each collection site), lab negatives (unused filter as a DNA extraction negative control), and positive controls (2 L of water filtered from the ZSCF breeding pen). We sprayed filtration cups and forceps with DNA-away surface decontaminant (Molecular Bioproducts, USA) and rinsed 3× with distilled water between samples. Once out of the field, we stored filters in a cool, dry room in a sterilized pack with silica gel.

### DNA extractions

We extracted DNA from every filter membrane independently following a modified Qiagen DNeasy Blood and Tissue Kit specifically for eDNA^55^. We transferred each membrane into a sterile Petri dish using single-use pipette tips and cut it into two halves using a sterile razor blade. We further cut each half into smaller pieces (~ 1 cm) before transferring them into separate 2.0 mL microcentrifuge tubes (one per half) containing approximately 250 g of 1 mm diameter glass beads. We then added 380 μL of ATL buffer (Qiagen) and put them into a TissueLyser (Qiagen) for 5 min at 25 Hz, after which they were spun down for 30 s at 11,000×*g*. We repeated the tissue disruption process twice for each subsample after inverting the tube holders. Next, we added 20 μL of Proteinase K, vortexed for 10 s, and spun down for 30 s at 11,000 g before incubating at 56 °C at 700 rpm overnight in a light duty orbital shaker (Ohaus). We vortexed for 15 s and centrifuged for 30 s at 11,000×*g* again. We added 400 μL of buffer AL, vortexed for 10 s, and incubated at 56 °C for 10 min. Following incubation, we added 400 μL of 95–100% ethanol and briefly vortexed. We transferred 640 μL of the mixture into a DNeasy Spin Column, settled in a 2 mL collection tube, centrifuged for 1 min at 11,000×*g* and repeated this step twice until transferring all the lysate. We added 500 μL of buffer AW1 (Qiagen) to wash the material retained on the silica membrane of the DNeasy Spin Column, and replaced the collection tube with a new 2 mL tube, after centrifuging for 1 min at 11,000×*g*. We washed the membrane again with 500 μL of buffer AW2 (Qiagen) and centrifuged for 5 min at 17,000×*g*. We carefully transferred the Spin Columns to a LoBind microcentrifuge tube (LoBind Eppendorf), added 200 μL of 70 °C prewarmed buffer AE (Qiagen), incubated for 15 min at room temperature and centrifuged for 5 min at 11,000×*g*. We stored the LoBind tubes with DNA extract at − 20 °C. We performed a second elution step using 100 μL of prewarmed buffer AE to collect a reserve elution, which we stored in LoBind tubes at − 20 °C. Before amplification, we created a master DNA extract tube per sample by pooling across the extract products of each filter membrane that resulted from each 1 L sample. This resulted in 49 master extracts being amplified (two for each of the 15 sampling sites plus the positive control pond, one for each field negative control, and one lab negative control).

### PCR amplification, library preparation, and MiSeq sequencing

We amplified all extract products in triplicates with four different PCR primer sets (Table 2). First, we tested Croc_COI^36^ and Croc_CR^14,56^, which amplify 548 and 458 bp fragments, respectively, of the mitochondrial cytochrome c oxidase subunit I (COI) and control region (CR). These markers were developed and used as part of several prior crocodylian population genetic and phylogenetic studies and represent the most common haplotype reference sequences in public databases for Neotropical *Crocodylus* species^14,36,56–59^. Second, we tested VertCOI^60,61^, a combination of versatile primer cocktails that amplify a 185 bp fragment of the COI mtDNA region with seven nucleotide substitutions between reported *C. rhombifer* and Cuban American crocodile haplotypes and that has been used widely in our lab in other eDNA metabarcoding studies (unpublished data). We additionally used VertCOI to simultaneously detect and describe the complement of biodiversity to be found at these sites, including available crocodylian prey. Finally, we used the MiFish primers^25^, which amplify a 220 bp fragment of the mitochondrial 12S gene region, to increase the success of fish detection as part of the available diet to crocodylians in Zapata Swamp.

**Table 2:** List of primers used in the present study (without including the Illumina adapters^67^) and thermocycling conditions followed during the first PCRs for eDNA metabarcoding library preparation for each primer set.

Target region	Primer (5ʹ–3ʹ)	Thermocycling conditions	References
Croc_COI	COIa: AGTATAAGCGTCTGGGTAGTC	Croc_COI: 94 °C (120 s) followed by 35 cycles at 94 °C (45 s), 48 °C (45 s), 72 °C (90 s); and a final extension of 72 °C (10 min)	^36,37^
	COIf: CCTGCAGGAGGAGGAGAYCC		
Croc_CR	drL15459: AGGAAAGCGCTGGCCTTGTAA	Croc_CR: 94 °C (120 s) followed by 33 cycles at 94 °C (30 s), 58 °C (30 s), 72 °C (45 s); and a final extension of 72 °C (7 min)	^56^
	CR2HA: GGGGCCACTAAAAACTGGGGGGA		
MiFish (12 S)	F: CCGGTAAAACTCGTGCCAGC	12S: 95 °C (180 s), 35 cycles of 98 °C (20 s), 65 °C (15 s), 72 °C (15 s), and a final extension of 72 °C (5 min)	^25^
	R: CATAGTGGGGTATCTAATCCCAGTTTG		
VertCOI	BloodmealF1_t1: ACCACWATTATTAAYATAAARCCMC	VertCOI: 94 °C (120 s) followed by 60 cycles at 94 °C (40 s), 56 °C (40 s), 72 °C (30 s); and a final extension of 72 °C (5 min)	^61^
	BloodmealF2_t1: ACTACAGCAATTAACATAAAACCMC		^61^
	VR1_t1:TAGACTTCTGGGTGGCCAAAGAATCA		^60^
	VR1d_t1:TAGACTTCTGGGTGGCCRAARAAYCA		^60^
	VR1i_t1:TAGACTTCTGGGTGNCCNAANAANCA		^60^

From each master extract tube, we amplified three technical replicates. We conducted PCRs in 25 μL reaction volumes containing 12.5 μL of 2× KAPA HiFi HS ReadyMix (Roche), 0.2 μM of primers with Illumina adaptors, and 2.5 μL of eDNA extract. We included a negative field control for every batch of amplified samples, and a sequencing blank (20 μL of molecular-grade water) was added to each plate previous to pooling and normalization of the libraries. Amplifications were accomplished with the thermocycling conditions indicated in Table 2. We amplified each sample a second time using the first PCR products as template after a clean-up using 1× NGS magnetic beads (Macherey–Nagel) following the manufacturer's protocol. In this round, we amplified the target regions using dual-index primer combinations for each sample where the sequence of index primers was equivalent to the Nextera XT Index Kit (Illumina). We performed round two PCRs in 50 μL reaction volumes, including 5 μL of cleaned PCR product, 5 μL of each index primer (10 μM), 25 μL of 2× KAPA HiFi, and 10 μL of molecular biology grade water. Cycling conditions were as follows: 95 °C (180 s), 12 cycles at 95 °C (30 s), 55 °C (30 s), 72 °C (30 s), and a final extension at 72 °C (300 s). We visualized second-round PCR products on a 2% agarose gel and then purified them using 1× NGS magnetic beads (Macherey–Nagel) following the manufacturer’s protocols. We sequenced each amplified region on an Illumina MiSeq System and reagent kit version 3 (600 cycles) at the Advanced Analysis Center (AAC) at the University of Guelph.

### Bioinformatics analysis

We uploaded raw FASTQ data files of CrocCOI and VertCOI to the Multiplex Barcode Research and Visualization Environmental (mBRAVE—Metabarcoding at Scale http://www.mbrave.net/). We analyzed the sequences using the platform algorithms for removing chimeras and assigning Operational Taxonomic Units (OTU) and BIN (Barcode Index Number) identification. Supplementary Information 2 shows the analytical parameters (trimming, filtering, paired-end, and other parameters) set for both COI primer sets. We identified reads to BINs against the following BOLD data sets: (1) SYS-MBRAVEC: System Reference Library—Standard Contaminants Based on Reagent Production; (2) SYS-HUMC: System Reference Library—Human Contamination Check; (3) SYS-CRLBACTERIA: System Reference Library for mBRAVE ID Engine—Bacteria COI; (4) SYS-CRLPROTISTA: System Reference Library for mBRAVE ID Engine—Protista COI; and (5) SYS-CRLCHORDATA: System Reference Library for mBRAVE ID Engine—Chordata.

We also used the bioinformatics software platform Geneious Prime version 2022.2.2 (https://www.geneious.com) to analyze the Croc_COI and Croc_CR FASTQ raw data files. We set the following parameter values: (1) Set paired reads: Pair by Pairs of sequence list, Relative Orientation: Forward/Reverse (inward), Expected Distance/Insert size: 665; (2) Trim using BBDuk—Trim adapters: All Truseq, Nextera and PhiX adapters (158 sequences), Trim: Right End, Kmer length: 27, Maximum substitutions: 1, Maximum substitution + INDELs: 0, Discard Short Reads: 200 bp minimum length; (3) Trim Ends—Trim primers sequences COIa and COIf, Allow mismatches: 5, Minimum match length: 5, Trim both 5ʹ and 3ʹ Ends, all new trimmed regions were removed; (4) Remove Duplicated Reads—it was performed on Dedupe Duplicate Read Remover 38.84 by Brian Bushnell, Kmer seed length: 31. We identified all reads against a local database built with all *C. rhombifer* and *C. acutus* sequences from Cuban crocodiles found on GenBank, and also including sequences from the following *Crocodylus* species (Genbank Accession Number): *C. intermedius* (HM636895), *C. johnsoni* (HM488008); *C mindorensis* (GU144287), *C. moreletii* (HQ585889); *C. niloticus* (AJ810452), *C. novaeguineae* (HM636896), *C. palustris* (GU144286), *C. porosus* (AJ810453), and *C. siamensis* (EF581859). We set the Basic Local Alignment Search Tool (BLAST) parameters as follows—Program: Megablast, Maximum hits: 1, Scoring mismatch: 1–2, Maximum E-value: 0.05, Maximum target sequence: 100.

We analyzed the fish mitochondrial 12S rRNA gene metabarcoding data using the MiFish pipeline (Mifish Pipeline (u-tokyo.ac.jp)). We uploaded paired reads to MiFish, where each pre-setting process was run (see^62^ for more details). We filtered the lists of species generated for each locality and sample leaving only the species with sequence similarity values ≥ 97% and a moderate or high confidence value.

Finally, we also used MetaWorks^63^, a multi-marker metabarcoding pipeline, to process paired-end Illumina reads from raw fastq.gz files of VertCOI and 12S amplified regions to taxonomic assignments. We set the parameters as follows: raw read pairing by SEQPREP: Phred score quality cut off: 20, minimum overlap length between forward and reverse reads: 25 bp, maximum fraction of mismatches allowed in overlap: 0.02 and the minimum fraction of matching overlap: 0.90; primer trimming: minimum sequence length to retain after trimming primers—150 bp (12S) and 100 bp (VertCOI), Phred quality score cutoffs at the ends: “20,20”, error rate: 0.1, minimum adapter overlap: 3 and the maximum number of N’s: 3; denoising: VSEARCH_DENOISE: min size = 3. Finally, the denoised ESVs were taxonomically assigned using the RDP classifier against custom-trained dataset COI, 12S_fish, and 12S_vertebrate. We filtered the species list generated by MetaWorks for each locality and sample by bootstrap support (≥ 0.97). Additional filtration (e.g., taxonomic and geographical curation) were applied regardless of the bioinformatic pipeline used to maximize confidence and accuracy of the taxonomic assigments, considering an already standardized methodology^64^.

To test differences in species richness amongst sites with and without crocodile eDNA detection, we implemented an Two-Sample F-test for Variances^65^ (a = 0.05) on the number of total taxa detected irrespective of primer set, after assessing normality (Shapiro–Wilk test) and heteroskedasticity (Bartlett test) of the data. Class category was the only one that did not meet the normality criteria and a Wilcoxon test^66^ was used instead.

### Supplementary Information

Supplementary Information 1.

Supplementary Information 2.

## Data Availability

The datasets generated (Raw data [FASTQ files]) and/or analyzed during the current study were made available in the NCBI Sequence Read Archive (Accession PRJNA958192) and Borealis Research Data Repository (https://doi.org/10.5683/SP3/UAUV3Z).
