# Supplementary material for: Environmental DNA-based biomonitoring of Cuban Crocodylus and their accompanying vertebrate fauna from Zapata Swamp, Cuba
Source: Sci Rep. 2023 Nov 22;13:20438. doi: 10.1038/s41598-023-47675-8 (PMC10665403; doi:10.1038/s41598-023-47675-8)
Supplement: Supplementary file 1 — Supplementary Information 1. [file 41598_2023_47675_MOESM1_ESM.docx]

Supplementary information 1. List of reference mitochondrial haplotype sequences for cytochrome c oxidase subunit I COI and control region (CR) used for *Crocodylus* detection with the Geneious pipeline for metabarcoding data analysis.

>HQ595058.1 Crocodylus rhombifer voucher CrDL182 cytochrome c oxidase subunit I gene, partial cds; mitochondrial

CTTATCCTGCCAGGGTTTGGAATAATTTCCCACGTAATTACCTTCTACTCAAGCAAAAAAGAACCATTTG

GTTATATAGGAATAGTATGAGCCATAATGTCAATTGGCTTCCTTGGCTTCATTGTCTGAGCCCACCACAT

GTTTACAGTAGGAATAGATGTTGACACTCGAGCATACTTCACATCCGCCACAATGATTATCGCCATCCCC

ACTGGTGTAAAAGTATTCAGCTGATTAGCCACTATTTATGGGGGAGTAGTAAAATGACAAGCCCCCATGC

TATGAGCACTCGGCTTCATTTTCTTATTCACAGTAGGAGGACTAACAGGAATTGTACTAGCTAACTCGTC

ACTGGACATTATTCTCCACGACACCTACTACGTAGTAGCCCACTTCCACTATGTGCTATCTATGGGAGCA

GTATTCGCCATCATAAGCGGATTCACCCACTGATTCCCATTATTTACAGGATTCACCCTACACAGCACAT

GAACAAAAATTCAATTCATGATCATATTTACAGGTGTAAACCTAACCTTCTTCCCA

>HQ595057.1 Crocodylus rhombifer voucher CrDL179 cytochrome c oxidase subunit I gene, partial cds; mitochondrial

CTTATCCTGCCAGGGTTTGGAATAATTTCCCACGTAATTACCTTCTACTCAAGCAAAAAAGAACCATTTG

GTTATATAGGAATAGTATGAGCCATAATGTCAATTGGCTTCCTTGGCTTCATTGTCTGAGCCCACCACAT

GTTTACAGTAGGAATAGATGTTGACACTCGAGCATACTTCACATCCGCCACAATGATTATCGCCATCCCC

ACTGGTGTAAAAGTATTCAGCTGATTAGCCACTATTTATGGGGGAGTAGTAAAATGACAAGCCCCCATGC

TATGAGCACTCGGCTTCATTTTCTTATTCACAGTAGGAGGACTAACAGGAATTGTACTAGCTAACTCGTC

ACTGGACATTATTCTCCACGACACCTACTACGTAGTAGCCCACTTCCACTATGTGCTATCTATGGGAGCA

GTATTCGCCATCATAAGCGGATTCACCCACTGATTCCCATTATTTACAGGATTCACCCTACACAGCACAT

GAACAAAAATTCAATTCATGATCATATTTACAGGTGTAAACCTAACCTTCTTCCCA

>HQ595056.1 Crocodylus rhombifer voucher CrCU7 cytochrome c oxidase subunit I gene, partial cds; mitochondrial

CTTATCCTGCCAGGGTTTGGAATAATTTCCCACGTAATTACCTTCTACTCAAGCAAAAAAGAACCATTTG

GTTATATAGGAATAGTATGAGCCATAATGTCAATTGGCTTCCTTGGCTTCATTGTCTGAGCCCACCACAT

GTTTACAGTAGGAATAGATGTTGACACTCGAGCATACTTCACATCCGCCACAATGATTATCGCCATCCCC

ACTGGTGTAAAAGTATTCAGCTGATTAGCCACTATTTATGGGGGAGTAGTAAAATGACAAGCCCCCATGC

TATGAGCACTCGGCTTCATTTTCTTATTCACAGTAGGAGGACTAACAGGAATTGTACTAGCTAACTCGTC

ACTGGACATTATTCTCCACGACACCTACTACGTAGTAGCCCACTTCCACTATGTGCTATCTATGGGAGCA

GTATTCGCCATCATAAGCGGATTCACCCACTGATTCCCATTATTTACAGGATTCACCCTACACAGCACAT

GAACAAAAATTCAATTCATGATCATATTTACAGGTGTAAACCTAACCTTCTTCCCA

>HQ595055.1 Crocodylus rhombifer voucher CrCU26 cytochrome c oxidase subunit I gene, partial cds; mitochondrial

CTTATCCTGCCAGGGTTTGGAATAATTTCCCACGTAATTACCTTCTACTCAAGCAAAAAAGAACCATTTG

GTTATATAGGAATAGTATGAGCCATAATGTCAATTGGCTTCCTTGGCTTCATTGTCTGAGCCCACCACAT

GTTTACAGTAGGAATAGATGTTGACACTCGAGCATACTTCACATCCGCCACAATGATTATCGCCATCCCC

ACTGGTGTAAAAGTATTCAGCTGATTAGCCACTATTTATGGGGGAGTAGTAAAATGACAAGCCCCCATGC

TATGAGCACTCGGCTTCATTTTCTTATTCACAGTAGGAGGACTAACAGGAATTGTACTAGCTAACTCGTC

ACTGGACATTATTCTCCACGACACCTACTACGTAGTAGCCCACTTCCACTATGTGCTATCTATGGGAGCA

GTATTCGCCATCATAAGCGGATTCACCCACTGATTCCCATTATTTACAGGATTCACCCTACACAGCACAT

GAACAAAAATTCAATTCATGATCATATTTACAGGTGTAAACCTAACCTTCTTCCCA

>HQ595054.1 Crocodylus rhombifer voucher CrCU25 cytochrome c oxidase subunit I gene, partial cds; mitochondrial

CTTATCCTGCCAGGGTTTGGAATAATTTCCCACGTAATTACCTTCTACTCAAGCAAAAAAGAACCATTTG

GTTATATAGGAATAGTATGAGCCATAATGTCAATTGGCTTCCTTGGCTTCATTGTCTGAGCCCACCACAT

GTTTACAGTAGGAATAGATGTTGACACTCGAGCATACTTCACATCCGCCACAATGATTATCGCCATCCCC

ACTGGTGTAAAAGTATTCAGCTGATTAGCCACTATTTATGGGGGAGTAGTAAAATGACAAGCCCCCATGC

TATGAGCACTCGGCTTCATTTTCTTATTCACAGTAGGAGGACTAACAGGAATTGTACTAGCTAACTCGTC

ACTGGACATTATTCTCCACGACACCTACTACGTAGTAGCCCACTTCCACTATGTGCTATCTATGGGAGCA

GTATTCGCCATCATAAGCGGATTCACCCACTGATTCCCATTATTTACAGGATTCACCCTACACAGCACAT

GAACAAAAATTCAATTCATGATCATATTTACAGGTGTAAACCTAACCTTCTTCCCA

>HQ595053.1 Crocodylus rhombifer voucher CrCU22 cytochrome c oxidase subunit I gene, partial cds; mitochondrial

CTTATCCTGCCAGGGTTTGGAATAATTTCCCACGTAATTACCTTCTACTCAAGCAAAAAAGAACCATTTG

GTTATATAGGAATAGTATGAGCCATAATGTCAATTGGCTTCCTTGGCTTCATTGTCTGAGCCCACCACAT

GTTTACAGTAGGAATAGATGTTGACACTCGAGCATACTTCACATCCGCCACAATGATTATCGCCATCCCC

ACTGGTGTAAAAGTATTCAGCTGATTAGCCACTATTTATGGGGGAGTAGTAAAATGACAAGCCCCCATGC

TATGAGCACTCGGCTTCATTTTCTTATTCACAGTAGGAGGACTAACAGGAATTGTACTAGCTAACTCGTC

ACTGGACATTATTCTCCACGACACCTACTACGTAGTAGCCCACTTCCACTATGTGCTATCTATGGGAGCA

GTATTCGCCATCATAAGCGGATTCACCCACTGATTCCCATTATTTACAGGATTCACCCTACACAGCACAT

GAACAAAAATTCAATTCATGATCATATTTACAGGTGTAAACCTAACCTTCTTCCCA

>HQ595052.1 Crocodylus rhombifer voucher CrCU21 cytochrome c oxidase subunit I gene, partial cds; mitochondrial

CTTATCCTGCCAGGGTTTGGAATAATTTCCCACGTAATTACCTTCTACTCAAGCAAAAAAGAACCATTTG

GTTATATAGGAATAGTATGAGCCATAATGTCAATTGGCTTCCTTGGCTTCATTGTCTGAGCCCACCACAT

GTTTACAGTAGGAATAGATGTTGACACTCGAGCATACTTCACATCCGCCACAATGATTATCGCCATCCCC

ACTGGTGTAAAAGTATTCAGCTGATTAGCCACTATTTATGGGGGAGTAGTAAAATGACAAGCCCCCATGC

TATGAGCACTCGGCTTCATTTTCTTATTCACAGTAGGAGGACTAACAGGAATTGTACTAGCTAACTCGTC

ACTGGACATTATTCTCCACGACACCTACTACGTAGTAGCCCACTTCCACTATGTGCTATCTATGGGAGCA

GTATTCGCCATCATAAGCGGATTCACCCACTGATTCCCATTATTTACAGGATTCACCCTACACAGCACAT

GAACAAAAATTCAATTCATGATCATATTTACAGGTGTAAACCTAACCTTCTTCCCA

>HQ595051.1 Crocodylus rhombifer voucher CrCU19 cytochrome c oxidase subunit I gene, partial cds; mitochondrial

CTTATCCTGCCAGGGTTTGGAATAATTTCCCACGTAATTACCTTCTACTCAAGCAAAAAAGAACCATTTG

GTTATATAGGAATAGTATGAGCCATAATGTCAATTGGCTTCCTTGGCTTCATTGTCTGAGCCCACCACAT

GTTTACAGTAGGAATAGATGTTGACACTCGAGCATACTTCACATCCGCCACAATGATTATCGCCATCCCC

ACTGGTGTAAAAGTATTCAGCTGATTAGCCACTATTTATGGGGGAGTAGTAAAATGACAAGCCCCCATGC

TATGAGCACTCGGCTTCATTTTCTTATTCACAGTAGGAGGACTAACAGGAATTGTACTAGCTAACTCGTC

ACTGGACATTATTCTCCACGACACCTACTACGTAGTAGCCCACTTCCACTATGTGCTATCTATGGGAGCA

GTATTCGCCATCATAAGCGGATTCACCCACTGATTCCCATTATTTACAGGATTCACCCTACACAGCACAT

GAACAAAAATTCAATTCATGATCATATTTACAGGTGTAAACCTAACCTTCTTCCCA

>HQ595050.1 Crocodylus rhombifer voucher CrCU15 cytochrome c oxidase subunit I gene, partial cds; mitochondrial

CTTATCCTGCCAGGGTTTGGAATAATTTCCCACGTAATTACCTTCTACTCAAGCAAAAAAGAACCATTTG

GTTATATAGGAATAGTATGAGCCATAATGTCAATTGGCTTCCTTGGCTTCATTGTCTGAGCCCACCACAT

GTTTACAGTAGGAATAGATGTTGACACTCGAGCATACTTCACATCCGCCACAATGATTATCGCCATCCCC

ACTGGTGTAAAAGTATTCAGCTGATTAGCCACTATTTATGGGGGAGTAGTAAAATGACAAGCCCCCATGC

TATGAGCACTCGGCTTCATTTTCTTATTCACAGTAGGAGGACTAACAGGAATTGTACTAGCTAACTCGTC

ACTGGACATTATTCTCCACGACACCTACTACGTAGTAGCCCACTTCCACTATGTGCTATCTATGGGAGCA

GTATTCGCCATCATAAGCGGATTCACCCACTGATTCCCATTATTTACAGGATTCACCCTACACAGCACAT

GAACAAAAATTCAATTCATGATCATATTTACAGGTGTAAACCTAACCTTCTTCCCA

>HQ595049.1 Crocodylus rhombifer voucher CrCU10 cytochrome c oxidase subunit I gene, partial cds; mitochondrial

CTTATCCTGCCAGGGTTTGGAATAATTTCCCACGTAATTACCTTCTACTCAAGCAAAAAAGAACCATTTG

GTTATATAGGAATAGTATGAGCCATAATGTCAATTGGCTTCCTTGGCTTCATTGTCTGAGCCCACCACAT

GTTTACAGTAGGAATAGATGTTGACACTCGAGCATACTTCACATCCGCCACAATGATTATCGCCATCCCC

ACTGGTGTAAAAGTATTCAGCTGATTAGCCACTATTTATGGGGGAGTAGTAAAATGACAAGCCCCCATGC

TATGAGCACTCGGCTTCATTTTCTTATTCACAGTAGGAGGACTAACAGGAATTGTACTAGCTAACTCGTC

ACTGGACATTATTCTCCACGACACCTACTACGTAGTAGCCCACTTCCACTATGTGCTATCTATGGGAGCA

GTATTCGCCATCATAAGCGGATTCACCCACTGATTCCCATTATTTACAGGATTCACCCTACACAGCACAT

GAACAAAAATTCAATTCATGATCATATTTACAGGTGTAAACCTAACCTTCTTCCCA

>MH273687.1 Crocodylus rhombifer voucher USNM:Herp:544376 cytochrome oxidase subunit 1 (COI) gene, partial cds; mitochondrial

CTTGTACTTTATTTTCGGCGCCTGAGCCGGAATAGTAGGCACAGCCATAAGCCTATTAATCCGAACAGAG

CTCAGCCAGCCGGGTCCCTTCATAGGAGATGACCAAATTTACAATGTCATTGTTACAGCACATGCCTTTA

TCATAATTTTCTTTATAGTTATACCAATCATGATCGGGGGATTTGGAAATTGACTACTCCCATTAATAAT

TGGAGCACCCGACATAGCATTCCCTCGCATAAACAACATAAGCTTCTGATTGCTGCCCCCATCATTTACC

CTACTCCTCTTTTCCGCCTTTATTGAAACTGGGGCTGGCACCGGATGAACAGTCTACCCACCTCTAGCTG

GAAACCTAGCCCACGCCGGACCATCAGTAGACCTCACTATCTTCTCCCTTCACCTTGCTGGAGTGTCATC

CATCCTTGGAGCAATTAATTTTATTACCACAGCTATCAACATAAAACCCCCAGCAATGTCACAACAACAA

ACGCCCCTTTTTGTATGATCTGTTCTAGTTACAGCTGTTCTCCTATTGCTATCACTACCCGTCCTAGCTG

CAGGGATTACTATATTACTCACTGACCGAAACTTGAACACCACTTTCTTCGACCCGGCAGGAGGAGGAGA

CCCAATCCTATACCAACACCTTTTC

>MH273686.1 Crocodylus rhombifer voucher USNM:Herp:544377 cytochrome oxidase subunit 1 (COI) gene, partial cds; mitochondrial

CTTGTACTTTATTTTCGGCGCCTGAGCCGGAATAGTAGGCACAGCCATAAGCCTATTAATCCGAACAGAG

CTCAGCCAGCCGGGTCCCTTCATAGGAGATGACCAAATTTACAATGTCATTGTTACAGCACATGCCTTTA

TCATAATTTTCTTTATAGTTATACCAATCATGATCGGGGGATTTGGAAATTGACTACTCCCATTAATAAT

TGGAGCACCCGACATAGCATTCCCTCGCATAAACAACATAAGCTTCTGATTGCTGCCCCCATCATTTACC

CTACTCCTCTTTTCCGCCTTTATTGAAACTGGGGCTGGCACCGGATGAACAGTCTACCCACCTCTAGCTG

GAAACCTAGCCCACGCCGGACCATCAGTAGACCTCACTATCTTCTCCCTTCACCTTGCTGGAGTGTCATC

CATCCTTGGAGCAATTAATTTTATTACCACAGCTATCAACATAAAACCCCCAGCAATGTCACAACAACAA

ACGCCCCTTTTTGTATGATCTGTTCTAGTTACAGCTGTTCTCCTATTGCTATCACTACCCGTCCTAGCTG

CAGGGATTACTATATTACTCACTGACCGAAACTTGAACACCACTTTCTTCGACCCGGCAGGAGGAGGAGA

CCCAATCCTATACCAACACCTTTTC

>HQ595048.1 Crocodylus rhombifer x acutus voucher CrambCU12 cytochrome c oxidase subunit I gene, partial cds; mitochondrial

CTTATCCTGCCAGGGTTTGGAATAATTTCCCACGTAATTACCTTCTACTCAAGCAAAAAAGAACCATTTG

GTTATATAGGAATAGTATGAGCCATAATGTCAATTGGCTTCCTTGGCTTCATTGTCTGAGCCCACCACAT

GTTTACAGTAGGAATAGATGTTGACACTCGAGCATACTTCACATCCGCCACAATGATTATCGCCATCCCC

ACTGGTGTAAAAGTATTCAGCTGATTAGCCACTATTTATGGGGGAGTAGTAAAATGACAAGCCCCCATGC

TATGAGCACTCGGCTTCATTTTCTTATTCACAGTAGGAGGACTAACAGGAATTGTACTAGCTAACTCGTC

ACTGGACATTATTCTCCACGACACCTACTACGTAGTAGCCCACTTCCACTATGTGCTATCTATGGGAGCA

GTATTCGCCATCATAAGCGGATTCACCCACTGATTCCCATTATTTACAGGATTCACCCTACACAGCACAT

GAACAAAAATTCAATTCATGATCATATTTACAGGTGTAAACCTAACCTTCTTCCCA

>HQ595047.1 Crocodylus rhombifer x acutus voucher CrambCU11 cytochrome c oxidase subunit I gene, partial cds; mitochondrial

CTTATCCTGCCAGGGTTTGGAATAATTTCCCACGTAATTACCTTCTACTCAAGCAAAAAAGAACCATTTG

GTTATATAGGAATAGTATGAGCCATAATGTCAATTGGCTTCCTTGGCTTCATTGTCTGAGCCCACCACAT

GTTTACAGTAGGAATAGATGTTGACACTCGAGCATACTTCACATCCGCCACAATGATTATCGCCATCCCC

ACTGGTGTAAAAGTATTCAGCTGATTAGCCACTATTTATGGGGGAGTAGTAAAATGACAAGCCCCCATGC

TATGAGCACTCGGCTTCATTTTCTTATTCACAGTAGGAGGACTAACAGGAATTGTACTAGCTAACTCGTC

ACTGGACATTATTCTCCACGACACCTACTACGTAGTAGCCCACTTCCACTATGTGCTATCTATGGGAGCA

GTATTCGCCATCATAAGCGGATTCACCCACTGATTCCCATTATTTACAGGATTCACCCTACACAGCACAT

GAACAAAAATTCAATTCATGATCATATTTACAGGTGTAAACCTAACCTTCTTCCCA

>HQ595027.1 Crocodylus acutus voucher CaCA50 cytochrome c oxidase subunit I gene, partial cds; mitochondrial

CTTATCCTGCCAGGGTTTGGAATAATCTCCCACGTAATTACATTCTACTCAAGCAAAAAAGAACCATTTG

GTTATATGGGGATAGTCTGAGCCATAATGTCAATCGGCTTCCTTGGATTCATTGTCTGAGCCCACCACAT

GTTTACAGTAGGGATAGATGTTGATACTCGAGCATATTTCACATCCGCCACAATAATTATCGCCATCCCC

ACTGGTGTAAAAGTATTCAGCTGATTAGCCACTATTTACGGAGGGGTAGTAAAATGACAAGCCCCCATGC

TCTGAGCACTCGGCTTCATTTTCTTATTCACAGTTGGAGGACTAACAGGAATTGTACTAGCTAACTCGTC

ACTAGACATTATTCTCCACGATACCTACTACGTAGTAGCCCACTTCCACTATGTATTATCTATGGGAGCA

GTGTTCGCCATCATAAGCGGGTTCACCCACTGATTCCCGCTATTCACAGGATTCACCCTACACAGCACAT

GAACAAAAATTCAATTCATAATCATATTTACAGGTGTAAATCTAACCTTCTTCCCA

>HQ595028.1 Crocodylus acutus voucher CaCA51 cytochrome c oxidase subunit I gene, partial cds; mitochondrial

CTTATCCTGCCAGGGTTTGGAATAATCTCCCACGTAATTACATTCTACTCAAGCAAAAAAGAACCATTTG

GTTATATGGGGATAGTCTGAGCCATAATGTCAATCGGCTTCCTTGGATTCATTGTCTGAGCCCACCACAT

GTTTACAGTAGGGATAGATGTTGATACTCGAGCATATTTCACATCCGCCACAATAATTATCGCCATCCCC

ACTGGTGTAAAAGTATTCAGCTGATTAGCCACTATTTACGGAGGGGTAGTAAAATGACAAGCCCCCATGC

TCTGAGCACTCGGCTTCATTTTCTTATTCACAGTTGGAGGACTAACAGGAATTGTACTAGCTAACTCGTC

ACTAGACATTATTCTCCACGATACCTACTACGTAGTAGCCCACTTCCACTATGTATTATCTATGGGAGCA

GTGTTCGCCATCATAAGCGGGTTCACCCACTGATTCCCGCTATTCACAGGATTCACCCTACACAGCACAT

GAACAAAAATTCAATTCATAATCATATTTACAGGTGTAAATCTAACCTTCTTCCCA

>HQ595029.1 Crocodylus acutus voucher CaCA52 cytochrome c oxidase subunit I gene, partial cds; mitochondrial

CTTATCCTGCCAGGGTTTGGAATAATCTCCCACGTAATTACATTCTACTCAAGCAAAAAAGAACCATTTG

GTTATATGGGGATAGTCTGAGCCATAATGTCAATCGGCTTCCTTGGATTCATTGTCTGAGCCCACCACAT

GTTTACAGTAGGGATAGATGTTGATACTCGAGCATATTTCACATCCGCCACAATAATTATCGCCATCCCC

ACTGGTGTAAAAGTATTCAGCTGATTAGCCACTATTTACGGAGGGGTAGTAAAATGACAAGCCCCCATGC

TCTGAGCACTCGGCTTCATTTTCTTATTCACAGTTGGAGGACTAACAGGAATTGTACTAGCTAACTCGTC

ACTAGACATTATTCTCCACGATACCTACTACGTAGTAGCCCACTTCCACTATGTATTATCTATGGGAGCA

GTGTTCGCCATCATAAGCGGGTTCACCCACTGATTCCCGCTATTCACAGGATTCACCCTACACAACACAT

GAACAAAAATTCAATTCATAATCATATTTACAGGTGTAAATCTAACCTTCTTCCCA

>HQ595030.1 Crocodylus acutus voucher CaCA53 cytochrome c oxidase subunit I gene, partial cds; mitochondrial

CTTATCCTGCCAGGGTTTGGAATAATCTCCCACGTAATTACATTCTACTCAAGCAAAAAAGAACCATTTG

GTTATATGGGGATAGTCTGAGCCATAATGTCAATCGGCTTCCTTGGATTCATTGTCTGAGCCCACCACAT

GTTTACAGTAGGGATAGATGTTGATACTCGAGCATATTTCACATCCGCCACAATAATTATCGCCATCCCC

ACTGGTGTAAAAGTATTCAGCTGATTAGCCACTATTTACGGAGGGGTAGTAAAATGACAAGCCCCCATGC

TCTGAGCACTCGGCTTCATTTTCTTATTCACAGTTGGAGGACTAACAGGAATTGTACTAGCTAACTCGTC

ACTAGACATTATTCTCCACGATACCTACTACGTAGTAGCCCACTTCCACTATGTATTATCTATGGGAGCA

GTGTTCGCCATCATAAGCGGGTTCACCCACTGATTCCCGCTATTCACAGGATTCACCCTACACAGCACAT

GAACAAAAATTCAATTCATAATCATATTTACAGGTGTAAATCTAACCTTCTTCCCA

>HQ595031.1 Crocodylus acutus voucher CaCA54 cytochrome c oxidase subunit I gene, partial cds; mitochondrial

CTTATCCTGCCAGGGTTTGGAATAATCTCCCACGTAATTACATTCTACTCAAGCAAAAAAGAACCATTTG

GTTATATGGGGATAGTCTGAGCCATAATGTCAATCGGCTTCCTTGGATTCATTGTCTGAGCCCACCACAT

GTTTACAGTAGGGATAGATGTTGATACTCGAGCATATTTCACATCCGCCACAATAATTATCGCCATCCCC

ACTGGTGTAAAAGTATTCAGCTGATTAGCCACTATTTACGGAGGGGTAGTAAAATGACAAGCCCCCATGC

TCTGAGCACTCGGCTTCATTTTCTTATTCACAGTCGGAGGACTAACAGGAATTGTACTAGCTAACTCGTC

ACTAGACATTATTCTCCACGATACCTACTACGTAGTAGCCCACTTCCACTATGTATTATCTATGGGAGCA

GTGTTCGCCATCATAAGCGGGTTCACCCACTGATTCCCGCTATTCACAGGATTCACCCTACACAGCACAT

GAACAAAAATTCAATTCATAATCATATTTACAGGTGTAAATCTAACCTTCTTCCCA

>HQ595032.1 Crocodylus acutus voucher CaCA55 cytochrome c oxidase subunit I gene, partial cds; mitochondrial

CTTATCCTGCCAGGGTTTGGAATAATCTCCCACGTAATTACATTCTACTCAAGCAAAAAAGAACCATTTG

GTTATATGGGGATAGTCTGAGCCATAATGTCAATCGGCTTCCTTGGATTCATTGTCTGAGCCCACCACAT

GTTTACAGTAGGGATAGATGTTGATACTCGAGCATATTTCACATCCGCCACAATAATTATCGCCATCCCC

ACTGGTGTAAAAGTATTCAGCTGATTAGCCACTATTTACGGAGGGGTAGTAAAATGACAAGCCCCCATGC

TCTGAGCACTCGGCTTCATTTTCTTATTCACAGTTGGAGGACTAACAGGAATTGTACTAGCTAACTCGTC

ACTAGACATTATTCTCCACGATACCTACTACGTAGTAGCCCACTTCCACTATGTATTATCTATGGGAGCA

GTGTTCGCCATCATAAGCGGGTTCACCCACTGATTCCCGCTATTCACAGGATTCACCCTACACAGCACAT

GAACAAAAATTCAATTCATAATCATATTTACAGGTGTAAATCTAACCTTCTTCCCA

>HQ595033.1 Crocodylus acutus voucher CaCA56 cytochrome c oxidase subunit I gene, partial cds; mitochondrial

CTTATCCTGCCAGGGTTTGGAATAATCTCCCACGTAATTACATTCTACTCAAGCAAAAAAGAACCATTTG

GTTATATGGGGATAGTCTGAGCCATAATGTCAATCGGCTTCCTTGGATTCATTGTCTGAGCCCACCACAT

GTTTACAGTAGGGATAGATGTTGATACTCGAGCATATTTCACATCCGCCACAATAATTATCGCCATCCCC

ACTGGTGTAAAAGTATTCAGCTGATTAGCCACTATTTACGGAGGGGTAGTAAAATGACAAGCCCCCATGC

TCTGAGCACTCGGCTTCATTTTCTTATTCACAGTCGGAGGACTAACAGGAATTGTACTAGCTAACTCGTC

ACTAGACATTATTCTCCACGATACCTACTACGTAGTAGCCCACTTCCACTATGTATTATCTATGGGAGCA

GTGTTCGCCATCATAAGCGGGTTCACCCACTGATTCCCGCTATTCACAGGATTCACCCTACACAGCACAT

GAACAAAAATTCAATTCATAATCATATTTACAGGTGTAAATCTAACCTTCTTCCCA

>HQ595034.1 Crocodylus acutus voucher CaCU17 cytochrome c oxidase subunit I gene, partial cds; mitochondrial

CTTATCCTGCCAGGGTTTGGAATAATTTCCCACGTAATTACCTTCTACTCAAGCAAAAAAGAACCATTTG

GTTATATAGGAATAGTATGAGCCATAATGTCAATTGGCTTCCTTGGCTTCATTGTCTGAGCCCACCACAT

GTTTACAGTAGGAATAGATGTTGACACTCGAGCATACTTCACATCCGCCACAATGATTATCGCCATCCCC

ACTGGTGTAAAAGTATTCAGCTGATTAGCCACTATTTATGGGGGAGTAGTAAAATGACAAGCCCCCATGC

TATGAGCACTCGGCTTCATTTTCTTATTCACAGTAGGAGGACTAACAGGAATTGTACTAGCTAACTCGTC

ACTGGACATTATTCTCCACGACACCTACTACGTAGTAGCCCACTTCCACTATGTACTATCTATGGGAGCA

GTATTCGCCATCATAAGCGGATTCACCCACTGATTCCCACTATTTACAGGATTCACCCTACACAGCACAT

GAACAAAAATTCAATTCATGATCATATTTACAGGTGTAAACCTAACCTTCTTCCCA

>HQ595035.1 Crocodylus acutus voucher CaCU18 cytochrome c oxidase subunit I gene, partial cds; mitochondrial

CTTATCCTGCCAGGGTTTGGAATAATTTCCCACGTAATTACCTTCTACTCAAGCAAAAAAGAACCATTTG

GTTATATAGGAATAGTATGAGCCATAATGTCAATTGGCTTCCTTGGCTTCATTGTCTGAGCCCACCACAT

GTTTACAGTAGGAATAGATGTTGACACTCGAGCATACTTCACATCCGCCACAATGATTATCGCCATCCCC

ACTGGTGTAAAAGTATTCAGCTGATTAGCCACTATTTATGGGGGAGTAGTAAAATGACAAGCCCCCATGC

TATGAGCACTCGGCTTCATTTTCTTATTCACAGTAGGAGGACTAACAGGAATTGTACTAGCTAACTCGTC

ACTGGACATTATTCTCCACGACACCTACTACGTAGTAGCCCACTTCCACTATGTACTATCTATGGGAGCA

GTATTCGCCATCATAAGCGGATTCACCCACTGATTCCCACTATTTACAGGATTCACCCTACACAGCACAT

GAACAAAAATTCAATTCATGATCATATTTACAGGTGTAAACCTAACCTTCTTCCCA

>HQ595036.1 Crocodylus acutus voucher CaCU19 cytochrome c oxidase subunit I gene, partial cds; mitochondrial

CTTATCCTGCCAGGGTTTGGAATAATTTCCCACGTAATTACCTTCTACTCAAGCAAAAAAGAACCATTTG

GTTATATAGGAATAGTATGAGCCATAATGTCAATTGGCTTCCTTGGCTTCATTGTCTGAGCCCACCACAT

GTTTACAGTAGGAATAGATGTTGACACTCGAGCATACTTCACATCCGCCACAATGATTATCGCCATCCCC

ACTGGTGTAAAAGTATTCAGCTGATTAGCCACTATTTATGGGGGAGTAGTAAAATGACAAGCCCCCATGC

TATGAGCACTCGGCTTCATTTTCTTATTCACAGTAGGAGGACTAACAGGAATTGTACTAGCTAACTCGTC

ACTGGACATTATTCTCCACGACACCTACTACGTAGTAGCCCACTTCCACTATGTACTATCTATGGGAGCA

GTATTCGCCATCATAAGCGGATTCACCCACTGATTCCCACTATTTACAGGATTCACCCTACACAGCACAT

GAACAAAAATTCAATTCATGATCATATTTACAGGTGTAAACCTAACCTTCTTCCCA

>HQ595037.1 Crocodylus acutus voucher CaCU21 cytochrome c oxidase subunit I gene, partial cds; mitochondrial

CTTATCCTGCCAGGGTTTGGAATAATTTCCCACGTAATTACCTTCTACTCAAGCAAAAAAGAACCATTTG

GTTATATAGGAATAGTATGAGCCATAATGTCAATTGGCTTCCTTGGCTTCATTGTCTGAGCCCACCACAT

GTTTACAGTAGGAATAGATGTTGACACTCGAGCATACTTCACATCCGCCACAATGATTATCGCCATCCCC

ACTGGTGTAAAAGTATTCAGCTGATTAGCCACTATTTATGGGGGAGTAGTAAAATGACAAGCCCCCATGC

TATGAGCACTCGGCTTCATTTTCTTATTCACAGTAGGAGGACTAACAGGAATTGTACTAGCTAACTCGTC

ACTGGACATTATTCTCCACGACACCTACTACGTAGTAGCCCACTTCCACTATGTACTATCTATGGGAGCA

GTATTCGCCATCATAAGCGGATTCACCCACTGATTCCCACTATTTACAGGATTCACCCTACACAGCACAT

GAACAAAAATTCAATTCATGATCATATTTACAGGTGTAAACCTAACCTTCTTCCCA

>HQ595038.1 Crocodylus acutus voucher CaCU22 cytochrome c oxidase subunit I gene, partial cds; mitochondrial

CTTATCCTGCCAGGGTTTGGAATAATTTCCCACGTAATTACCTTCTACTCAAGCAAAAAAGAACCATTTG

GTTATATAGGAATAGTATGAGCCATAATGTCAATTGGCTTCCTTGGCTTCATTGTCTGAGCCCACCACAT

GTTTACAGTAGGAATAGATGTTGACACTCGAGCATACTTCACATCCGCCACAATGATTATCGCCATCCCC

ACTGGTGTAAAAGTATTCAGCTGATTAGCCACTATTTATGGGGGAGTAGTAAAATGACAAGCCCCCATGC

TATGAGCACTCGGCTTCATTTTCTTATTCACAGTAGGAGGACTAACAGGAATTGTACTAGCTAACTCGTC

ACTGGACATTATTCTCCACGACACCTACTACGTAGTAGCCCACTTCCACTATGTACTATCTATGGGAGCA

GTATTCGCCATCATAAGCGGATTCACCCACTGATTCCCACTATTTACAGGATTCACCCTACACAGCACAT

GAACAAAAATTCAATTCATGATCATATTTACAGGTGTAAACCTAACCTTCTTCCCA

>HQ595039.1 Crocodylus acutus voucher CaCU23 cytochrome c oxidase subunit I gene, partial cds; mitochondrial

CTTATCCTGCCAGGGTTTGGAATAATTTCCCACGTAATTACCTTCTACTCAAGCAAAAAAGAACCATTTG

GTTATATAGGAATAGTATGAGCCATAATGTCAATTGGCTTCCTTGGCTTCATTGTCTGAGCCCACCACAT

GTTTACAGTAGGAATAGATGTTGACACTCGAGCATACTTCACATCCGCCACAATGATTATCGCCATCCCC

ACTGGTGTAAAAGTATTCAGCTGATTAGCCACTATTTATGGGGGAGTAGTAAAATGACAAGCCCCCATGC

TATGAGCACTCGGCTTCATTTTCTTATTCACAGTAGGAGGACTAACAGGAATTGTACTAGCTAACTCGTC

ACTGGACATTATTCTCCACGACACCTACTACGTAGTAGCCCACTTCCACTATGTACTATCTATGGGAGCA

GTATTCGCCATCATAAGCGGATTCACCCACTGATTCCCACTATTTACAGGATTCACCCTACACAGCACAT

GAACAAAAATTCAATTCATGATCATATTTACAGGTGTAAACCTAACCTTCTTCCCA

>HQ595040.1 Crocodylus acutus voucher CaCU37 cytochrome c oxidase subunit I gene, partial cds; mitochondrial

CTTATCCTGCCAGGGTTTGGAATAATTTCCCACGTAATTACCTTCTACTCAAGCAAAAAAGAACCATTTG

GTTATATAGGAATAGTATGAGCCATAATGTCAATTGGCTTCCTTGGCTTCATTGTCTGAGCCCACCACAT

GTTTACAGTAGGAATAGATGTTGACACTCGAGCATACTTCACATCCGCCACAATGATTATCGCCATCCCC

ACTGGTGTAAAAGTATTCAGCTGATTAGCCACTATTTATGGGGGAGTAGTAAAATGACAAGCCCCCATGC

TATGAGCACTCGGCTTCATTTTCTTATTCACAGTAGGAGGACTAACAGGAATTGTACTAGCTAACTCGTC

ACTGGACATTATTCTCCACGACACCTACTACGTAGTAGCCCACTTCCACTATGTACTATCTATGGGAGCA

GTATTCGCCATCATAAGCGGATTCACCCACTGATTCCCACTATTTACAGGATTCACCCTACACAGCACAT

GAACAAAAATTCAATTCATGATCATATTTACAGGTGTAAACCTAACCTTCTTCCCA

>HQ595041.1 Crocodylus acutus voucher CaJM347 cytochrome c oxidase subunit I gene, partial cds; mitochondrial

CTTATCCTGCCAGGGTTTGGAATAATTTCCCACGTAATTACCTTCTACTCAAGCAAAAAAGAACCATTTG

GTTATATAGGAATAGTATGAGCCATAATGTCAATTGGCTTCCTTGGCTTCATTGTCTGAGCCCACCACAT

GTTTACAGTAGGAATAGATGTTGACACTCGAGCATACTTCACATCCGCCACAATGATTATCGCCATCCCC

ACTGGTGTAAAAGTATTCAGCTGATTAGCCACTATTTATGGGGGAGTAGTAAAATGACAAGCCCCCATGC

TATGAGCACTCGGCTTCATTTTCTTATTCACAGTAGGAGGACTAACAGGAATTGTACTAGCTAACTCGTC

ACTGGACATTATTCTCCACGACACCTACTACGTAGTAGCCCACTTCCACTATGTACTATCTATGGGAGCA

GTATTCGCCATCATAAGCGGATTCACCCACTGATTCCCACTATTTACAGGATTCACCCTACACAGCACAT

GAACAAAAATTCAATTCATGATCATATTTACAGGTGTAAACCTAACCTTCTTCCCA

>HQ595042.1 Crocodylus acutus voucher CaJM348 cytochrome c oxidase subunit I gene, partial cds; mitochondrial

CTTATCCTGCCAGGGTTTGGAATAATTTCCCACGTAATTACCTTCTACTCAAGCAAAAAAGAACCATTTG

GTTATATAGGAATAGTATGAGCCATAATGTCAATTGGCTTCCTTGGCTTCATTGTCTGAGCCCACCACAT

GTTTACAGTAGGAATAGATGTTGACACTCGAGCATACTTCACATCCGCCACAATGATTATCGCCATCCCC

ACTGGTGTAAAAGTATTCAGCTGATTAGCCACTATTTATGGGGGAGTAGTAAAATGACAAGCCCCCATGC

TATGAGCACTCGGCTTCATTTTCTTATTCACAGTAGGAGGACTAACAGGAATTGTACTAGCTAACTCGTC

ACTGGACATTATTCTCCACGACACCTACTACGTAGTAGCCCACTTCCACTATGTACTATCTATGGGAGCA

GTATTCGCCATCATAAGCGGATTCACCCACTGATTCCCACTATTTACAGGATTCACCCTACACAGCACAT

GAACAAAAATTCAATTCATGATCATATTTACAGGTGTAAACCTAACCTTCTTCCCA

>HQ595043.1 Crocodylus acutus voucher CaKI48 cytochrome c oxidase subunit I gene, partial cds; mitochondrial

CTTATCCTGCCAGGGTTTGGAATAATTTCCCACGTAATTACCTTCTACTCAAGCAAAAAAGAACCATTTG

GTTATATAGGAATAGTATGAGCCATAATGTCAATTGGCTTCCTTGGCTTCATTGTCTGAGCCCACCACAT

GTTTACAGTAGGAATAGATGTTGACACTCGAGCATACTTCACATCCGCCACAATGATTATCGCCATCCCC

ACTGGTGTAAAAGTATTCAGCTGATTAGCCACTATTTATGGGGGAGTAGTAAAATGACAAGCCCCCATGC

TATGAGCACTCGGCTTCATTTTCTTATTCACAGTAGGAGGACTAACAGGAATTGTACTAGCTAACTCGTC

ACTGGACATTATTCTCCACGACACCTACTACGTAGTAGCCCACTTCCACTATGTACTATCTATGGGAGCA

GTATTCGCCATCATAAGCGGATTCACCCACTGATTCCCACTATTTACAGGATTCACCCTACACAGCACAT

GAACAAAAATTCAATTCATGATCATATTTACAGGTGTAAACCTAACCTTCTTCCCA

>EU034623.1 Crocodylus rhombifer strain MF1745 tRNA-Pro gene, partial sequence; tRNA-Phe gene, complete sequence; and D-loop, partial sequence; mitochondrial

CTTCTTGAACTAAATCAGTCATCGTAGCTTAATTCACAAAAGCATAACACTGAAAATGTTAATATGGACA

AAGAGTCCCGAATGACAAACAGACCAACCAAAAACCACGAGCACTATTACCTTCCCCCAAACAAAAAGAT

AATATGTACCACCCCCCAGCTATGTATTATAAGGCATTCATTTATCTGCCCCTAGCACCCATCCATTAGT

TCTTATTAGTCAGCATCTCACGTGAAATCACCATCCATTGTATCCATACTAACTATTACTAGTCTCAAGC

CCATACCTGGACACGGCTCACATTTATTGCTCTTCTTAGAGACCTCTGGTTATCACTCTCACGTACCCAT

CTTGCTATTGCCTGGACATTCTTTCCTCTTCTTAGAGGCCTCAACCCGCACCATATGGCTTCACTCATTC

ACGTCCGTAATCGCGGCATCCC

>EU034622.1 Crocodylus rhombifer strain MF1744 tRNA-Pro gene, partial sequence; tRNA-Phe gene, complete sequence; and D-loop, partial sequence; mitochondrial

CTTCTTGAACTAAATCAGTCATCGTAGCTTAATTCACAAAAGCATAACACTGAAAATGTTAATATGGACA

AAGAGTCCCGAATGACAAACAGACCAACCAAAAACCACGAGCACTATTACCTTCCCCCAAACAAAAAGAT

AATATGTACCACCCCCCAGCTATGTATTATAAGGCATTCATTTATCTGCCCCTAGCACCCATCCATTAGT

TCTTATTAGTCAGCATCTCACGTGAAATCACCATCCATTGTATCCATACTAACTATTACTAGTCTCAAGC

CCATACCTGGACACGGCTCACATTTATTGCTCTTCTTAGAGACCTCTGGTTATCACTCTCACGTACCCAT

CTTGCTATTGCCTGGACATTCTTTCCTCTTCTTAGAGGCCTCAACCCGCACCATATGGCTTCACTCATTC

ACGTCCGTAATCGCGGCATCCC

>EU034621.1 Crocodylus rhombifer strain MF277 tRNA-Pro gene, partial sequence; tRNA-Phe gene, complete sequence; and D-loop, partial sequence; mitochondrial

CTTCTTGAACTAAATCAGTCATCGTAGCTTAATTCACAAAAGCATAACACTGAAAATGTTAATATGGACA

AAGAGTCCCGAATGACAAACAGACCAACCAAAAACCACGAGCACTATTACCTTCCCCCAAACAAAAAGAT

AATATGTACCACCCCCCAGCTATGTATTATAAGGCATTCATTTATCTGCCCCTAGCACCCATCCATTAGT

TCTTATTAGTCAGCATCTCACGTGAAATCACCATCCATTGTATCCATACTAACTATTACTAGTCTCAAGC

CCATACCTGGACACGGCTCACATTTATTGCTCTTCTTAGAGACCTCTGGTTATCACTCTCACGTACCCAT

CTTGCTATTGCCTGGACATTCTTTCCTCTTCTTAGAGGCCTCAACCCGCACCATATGGCTTCACTCATTC

ACGTCCGTAATCGCGGCATCCC

>EU034620.1 Crocodylus rhombifer strain MF274 tRNA-Pro gene, partial sequence; tRNA-Phe gene, complete sequence; and D-loop, partial sequence; mitochondrial

CTTCTTGAACTAAATCAGTCATCGTAGCTTAATTCACAAAAGCATAACACTGAAAATGTTAATATGGACA

AAGAGTCCCGAATGACAAACAGACCAACCAAAAACCACGAGCACTATTACCTTCCCCCAAACAAAAAGAT

AATATGTACCACCCCCCAGCTATGTATTATAAGGCATTCATTTATCTGCCCCTAGCACCCATCCATTAGT

TCTTATTAGTCAGCATCTCACGTGAAATCACCATCCATTGTATCCATACTAACTATTACTAGTCTCAAGC

CCATACCTGGACACGGCTCACATTTATTGCTCTTCTTAGAGACCTCTGGTTATCACTCTCACGTACCCAT

CTTGCTATTGCCTGGACATTCTTTCCTCTTCTTAGAGGCCTCAACCCGCACCATATGGCTTCACTCATTC

ACGTCCGTAATCGCGGCATCCC

>EU034619.1 Crocodylus rhombifer strain MF272 tRNA-Pro gene, partial sequence; tRNA-Phe gene, complete sequence; and D-loop, partial sequence; mitochondrial

CTTCTTGAACTAAATCAGTCATCGTAGCTTAATTCACAAAAGCATAACACTGAAAATGTTAATATGGACA

AAGAGTCCCGAATGACAAACAGACCAACCAAAAACCACGAGCACTATTACCTTCCCCCAAACAAAAAGAT

AATATGTACCACCCCCCAGCTATGTATTATAAGGCATTCATTTATCTGCCCCTAGCACCCATCCATTAGT

TCTTATTAGTCAGCATCTCACGTGAAATCACCATCCATTGTATCCATACTAACTATTACTAGTCTCAAGC

CCATACCTGGACACGGCTCACATTTATTGCTCTTCTTAGAGACCTCTGGTTATCACTCTCACGTACCCAT

CTTGCTATTGCCTGGACATTCTTTCCTCTTCTTAGAGGCCTCAACCCGCACCATATGGCTTCACTCATTC

ACGTCCGTAATCGCGGCATCCC

>EU034618.1 Crocodylus rhombifer strain MF271 tRNA-Pro gene, partial sequence; tRNA-Phe gene, complete sequence; and D-loop, partial sequence; mitochondrial

CTTCTTGAACTAAATCAGTCATCGTAGCTTAATTCACAAAAGCATAACACTGAAAATGTTAATATGGACA

AAGAGTCCCGAATGACAAACAGACCAACCAAAAACCACGAGCACTATTACCTTCCCCCAAACAAAAAGAT

AATATGTACCACCCCCCAGCTATGTATTATAAGGCATTCATTTATCTGCCCCTAGCACCCATCCATTAGT

TCTTATTAGTCAGCATCTCACGTGAAATCACCATCCATTGTATCCATACTAACTATTACTAGTCTCAAGC

CCATACCTGGACACGGCTCACATTTATTGCTCTTCTTAGAGACCTCTGGTTATCACTCTCACGTACCCAT

CTTGCTATTGCCTGGACATTCTTTCCTCTTCTTAGAGGCCTCAACCCGCACCATATGGCTTCACTCATTC

ACGTCCGTAATCGCGGCATCCC

>EU034617.1 Crocodylus rhombifer strain MF270 tRNA-Pro gene, partial sequence; tRNA-Phe gene, complete sequence; and D-loop, partial sequence; mitochondrial

CTTCTTGAACTAAATCAGTCATCGTAGCTTAATTCACAAAAGCATAACACTGAAAATGTTAATATGGACA

AAGAGTCCCGAATGACAAACAGACCAACCAAAAACCACGAGCACTATTACCTTCCCCCAAACAAAAAGAT

AATATGTACCACCCCCCAGCTATGTATTATAAGGCATTCATTTATCTGCCCCTAGCACCCATCCATTAGT

TCTTATTAGTCAGCATCTCACGTGAAATCACCATCCATTGTATCCATACTAACTATTACTAGTCTCAAGC

CCATACCTGGACACGGCTCACATTTATTGCTCTTCTTAGAGACCTCTGGTTATCACTCTCACGTACCCAT

CTTGCTATTGCCTGGACATTCTTTCCTCTTCTTAGAGGCCTCAACCCGCACCATATGGCTTCACTCATTC

ACGTCCGTAATCGCGGCATCCC

>EU034616.1 Crocodylus rhombifer strain MF269 tRNA-Pro gene, partial sequence; tRNA-Phe gene, complete sequence; and D-loop, partial sequence; mitochondrial

CTTCTTGAACTAAATCAGTCATCGTAGCTTAATTCACAAAAGCATAACACTGAAAATGTTAATATGGACA

AAGAGTCCCGAATGACAAACAGACCAACCAAAAACCACGAGCACTATTACCTTCCCCCAAACAAAAAGAT

AATATGTACCACCCCCCAGCTATGTATTATAAGGCATTCATTTATCTGCCCCTAGCACCCATCCATTAGT

TCTTATTAGTCAGCATCTCACGTGAAATCACCATCCATTGTATCCATACTAACTATTACTAGTCTCAAGC

CCATACCTGGACACGGCTCACATTTATTGCTCTTCTTAGAGACCTCTGGTTATCACTCTCACGTACCCAT

CTTGCTATTGCCTGGACATTCTTTCCTCTTCTTAGAGGCCTCAACCCGCACCATATGGCTTCACTCATTC

ACGTCCGTAATCGCGGCATCCC

>EU034615.1 Crocodylus rhombifer strain MF268 tRNA-Pro gene, partial sequence; tRNA-Phe gene, complete sequence; and D-loop, partial sequence; mitochondrial

CTTCTTGAACTAAATCAGTCATCGTAGCTTAATTCACAAAAGCATAACACTGAAAATGTTAATATGGACA

AAGAGTCCCGAATGACAAACAGACCAACCAAAAACCACGAGCACTATTACCTTCCCCCAAACAAAAAGAT

AATATGTACCACCCCCCAGCTATGTATTATAAGGCATTCATTTATCTGCCCCTAGCACCCATCCATTAGT

TCTTATTAGTCAGCATCTCACGTGAAATCACCATCCATTGTATCCATACTAACTATTACTAGTCTCAAGC

CCATACCTGGACACGGCTCACATTTATTGCTCTTCTTAGAGACCTCTGGTTATCACTCTCACGTACCCAT

CTTGCTATTGCCTGGACATTCTTTCCTCTTCTTAGAGGCCTCAACCCGCACCATATGGCTTCACTCATTC

ACGTCCGTAATCGCGGCATCCC

>EU034614.1 Crocodylus rhombifer strain MF267 tRNA-Pro gene, partial sequence; tRNA-Phe gene, complete sequence; and D-loop, partial sequence; mitochondrial

CTTCTTGAACTAAATCAGTCATCGTAGCTTAATTCACAAAAGCATAACACTGAAAATGTTAATATGGACA

AAGAGTCCCGAATGACAAACAGACCAACCAAAAACCACGAGCACTATTACCTTCCCCCAAACAAAAAGAT

AATATGTACCACCCCCCAGCTATGTATTATAAGGCATTCATTTATCTGCCCCTAGCACCCATCCATTAGT

TCTTATTAGTCAGCATCTCACGTGAAATCACCATCCATTGTATCCATACTAACTATTACTAGTCTCAAGC

CCATACCTGGACACGGCTCACATTTATTGCTCTTCTTAGAGACCTCTGGTTATCACTCTCACGTACCCAT

CTTGCTATTGCCTGGACATTCTTTCCTCTTCTTAGAGGCCTCAACCCGCACCATATGGCTTCACTCATTC

ACGTCCGTAATCGCGGCATCCC

>EU034613.1 Crocodylus rhombifer strain LD324 tRNA-Pro gene, partial sequence; tRNA-Phe gene, complete sequence; and D-loop, partial sequence; mitochondrial

CTTCTTGAACTAAATCAGTCATCGTAGCTTAATTCACAAAAGCATAACACTGAAAATGTTAATATGGACA

AAGAGTCCCGAATGACAAACAGACCAACCAAAAACCACGAGCACTATTACCTTCCCCCAAACAAAAAGAT

AATATGTACCACCCCCCAGCTATGTATTATAAGGCATTCATTTATCTGCCCCTAGCACCCATCCATTAGT

TCTTATTAGTCAGCATCTCACGTGAAATCACCATCCATTGTATCCATACTAACTATTACTAGTCTCAAGC

CCATACCTGGACACGGCTCACATTTATTGCTCTTCTTAGAGACCTCTGGTTATCACTCTCACGTACCCAT

CTTGCTATTGCCTGGACATTCTTTCCTCTTCTTAGAGGCCTCAACCCGCACCATATGGCTTCACTCATTC

ACGTCCGTAATCGCGGCATCCC

>EU034612.1 Crocodylus rhombifer strain LD305 tRNA-Pro gene, partial sequence; tRNA-Phe gene, complete sequence; and D-loop, partial sequence; mitochondrial

CTTCTTGAACTAAATCAGTCATCGTAGCTTAATTCACAAAAGCATAACACTGAAAATGTTAATATGGACA

AAGAGTCCCGAATGACAAACAGACCAACCAAAAACCACGAGCACTATTACCTTCCCCCAAACAAAAAGAT

AATATGTACCACCCCCCAGCTATGTATTATAAGGCATTCATTTATCTGCCCCTAGCACCCATCCATTAGT

TCTTATTAGTCAGCATCTCACGTGAAATCACCATCCATTGTATCCATACTAACTATTACTAGTCTCAAGC

CCATACCTGGACACGGCTCACATTTATTGCTCTTCTTAGAGACCTCTGGTTATCACTCTCACGTACCCAT

CTTGCTATTGCCTGGACATTCTTTCCTCTTCTTAGAGGCCTCAACCCGCACCATATGGCTTCACTCATTC

ACGTCCGTAATCGCGGCATCCC

>EU034611.1 Crocodylus rhombifer strain LD221 tRNA-Pro gene, partial sequence; tRNA-Phe gene, complete sequence; and D-loop, partial sequence; mitochondrial

CTTCTTGAACTAAATCAGTCATCGTAGCTTAATTCACAAAAGCATAACACTGAAAATGTTAATATGGACA

AAGAGTCCCGAATGACAAACAGACCAACCAAAAACCACGAGCACTATTACCTTCCCCCAAACAAAAAGAT

AATATGTACCACCCCCCAGCTATGTATTATAAGGCATTCATTTATCTGCCCCTAGCACCCATCCATTAGT

TCTTATTAGTCAGCATCTCACGTGAAATCACCATCCATTGTATCCATACTAACTATTACTAGTCTCAAGC

CCATACCTGGACACGGCTCACATTTATTGCTCTTCTTAGAGACCTCTGGTTATCACTCTCACGTACCCAT

CTTGCTATTGCCTGGACATTCTTTCCTCTTCTTAGAGGCCTCAACCCGCACCATATGGCTTCACTCATTC

ACGTCCGTAATCGCGGCATCCC

>EU034610.1 Crocodylus rhombifer strain LD198 tRNA-Pro gene, partial sequence; tRNA-Phe gene, complete sequence; and D-loop, partial sequence; mitochondrial

CTTCTTGAACTAAATCAGTCATCGTAGCTTAATTCACAAAAGCATAACACTGAAAATGTTAATATGGACA

AAGAGTCCCGAATGACAAACAGACCAACCAAAAACCACGAGCACTATTACCTTCCCCCAAACAAAAAGAT

AATATGTACCACCCCCCAGCTATGTATTATAAGGCATTCATTTATCTGCCCCTAGCACCCATCCATTAGT

TCTTATTAGTCAGCATCTCACGTGAAATCACCATCCATTGTATCCATACTAACTATTACTAGTCTCAAGC

CCATACCTGGACACGGCTCACATTTATTGCTCTTCTTAGAGACCTCTGGTTATCACTCTCACGTACCCAT

CTTGCTATTGCCTGGACATTCTTTCCTCTTCTTAGAGGCCTCAACCCGCACCATATGGCTTCACTCATTC

ACGTCCGTAATCGCGGCATCCC

>EU034609.1 Crocodylus rhombifer strain LD197 tRNA-Pro gene, partial sequence; tRNA-Phe gene, complete sequence; and D-loop, partial sequence; mitochondrial

CTTCTTGAACTAAATCAGTCATCGTAGCTTAATTCACAAAAGCATAACACTGAAAATGTTAATATGGACA

AAGAGTCCCGAATGACAAACAGACCAACCAAAAACCACGAGCACTATTACCTTCCCCCAAACAAAAAGAT

AATATGTACCACCCCCCAGCTATGTATTATAAGGCATTCATTTATCTGCCCCTAGCACCCATCCATTAGT

TCTTATTAGTCAGCATCTCACGTGAAATCACCATCCATTGTATCCATACTAACTATTACTAGTCTCAAGC

CCATACCTGGACACGGCTCACATTTATTGCTCTTCTTAGAGACCTCTGGTTATCACTCTCACGTACCCAT

CTTGCTATTGCCTGGACATTCTTTCCTCTTCTTAGAGGCCTCAACCCGCACCATATGGCTTCACTCATTC

ACGTCCGTAATCGCGGCATCCC

>EU034608.1 Crocodylus rhombifer strain LD183 tRNA-Pro gene, partial sequence; tRNA-Phe gene, complete sequence; and D-loop, partial sequence; mitochondrial

CTTCTTGAACTAAATCAGTCATCGTAGCTTAATTCACAAAAGCATAACACTGAAAATGTTAATATGGACA

AAGAGTCCCGAATGACAAACAGACCAACCAAAAACCACGAGCACTATTACCTTCCCCCAAACAAAAAGAT

AATATGTACCACCCCCCAGCTATGTATTATAAGGCATTCATTTATCTGCCCCTAGCACCCATCCATTAGT

TCTTATTAGTCAGCATCTCACGTGAAATCACCATCCATTGTATCCATACTAACTATTACTAGTCTCAAGC

CCATACCTGGACACGGCTCACATTTATTGCTCTTCTTAGAGACCTCTGGTTATCACTCTCACGTACCCAT

CTTGCTATTGCCTGGACATTCTTTCCTCTTCTTAGAGGCCTCAACCCGCACCATATGGCTTCACTCATTC

ACGTCCGTAATCGCGGCATCCC

>EU034607.1 Crocodylus rhombifer strain LD182 tRNA-Pro gene, partial sequence; tRNA-Phe gene, complete sequence; and D-loop, partial sequence; mitochondrial

CTTCTTGAACTAAATCAGTCATCGTAGCTTAATTCACAAAAGCATAACACTGAAAATGTTAATATGGACA

AAGAGTCCCGAATGACAAACAGACCAACCAAAAACCACGAGCACTATTACCTTCCCCCAAACAAAAAGAT

AATATGTACCACCCCCCAGCTATGTATTATAAGGCATTCATTTATCTGCCCCTAGCACCCATCCATTAGT

TCTTATTAGTCAGCATCTCACGTGAAATCACCATCCATTGTATCCATACTAACTATTACTAGTCTCAAGC

CCATACCTGGACACGGCTCACATTTATTGCTCTTCTTAGAGACCTCTGGTTATCACTCTCACGTACCCAT

CTTGCTATTGCCTGGACATTCTTTCCTCTTCTTAGAGGCCTCAACCCGCACCATATGGCTTCACTCATTC

ACGTCCGTAATCGCGGCATCCC

>EU034606.1 Crocodylus rhombifer strain LD179 tRNA-Pro gene, partial sequence; tRNA-Phe gene, complete sequence; and D-loop, partial sequence; mitochondrial

CTTCTTGAACTAAATCAGTCATCGTAGCTTAATTCACAAAAGCATAACACTGAAAATGTTAATATGGACA

AAGAGTCCCGAATGACAAACAGACCAACCAAAAACCACGAGCACTATTACCTTCCCCCAAACAAAAAGAT

AATATGTACCACCCCCCAGCTATGTATTATAAGGCATTCATTTATCTGCCCCTAGCACCCATCCATTAGT

TCTTATTAGTCAGCATCTCACGTGAAATCACCATCCATTGTATCCATACTAACTATTACTAGTCTCAAGC

CCATACCTGGACACGGCTCACATTTATTGCTCTTCTTAGAGACCTCTGGTTATCACTCTCACGTACCCAT

CTTGCTATTGCCTGGACATTCTTTCCTCTTCTTAGAGGCCTCAACCCGCACCATATGGCTTCACTCATTC

ACGTCCGTAATCGCGGCATCCC

>EU034605.1 Crocodylus rhombifer strain LD178 tRNA-Pro gene, partial sequence; tRNA-Phe gene, complete sequence; and D-loop, partial sequence; mitochondrial

CTTCTTGAACTAAATCAGTCATCGTAGCTTAATTCACAAAAGCATAACACTGAAAATGTTAATATGGACA

AAGAGTCCCGAATGACAAACAGACCAACCAAAAACCACGAGCACTATTACCTTCCCCCAAACAAAAAGAT

AATATGTACCACCCCCCAGCTATGTATTATAAGGCATTCATTTATCTGCCCCTAGCACCCATCCATTAGT

TCTTATTAGTCAGCATCTCACGTGAAATCACCATCCATTGTATCCATACTAACTATTACTAGTCTCAAGC

CCATACCTGGACACGGCTCACATTTATTGCTCTTCTTAGAGACCTCTGGTTATCACTCTCACGTACCCAT

CTTGCTATTGCCTGGACATTCTTTCCTCTTCTTAGAGGCCTCAACCCGCACCATATGGCTTCACTCATTC

ACGTCCGTAATCGCGGCATCCC

>EU034604.1 Crocodylus rhombifer strain LD154 tRNA-Pro gene, partial sequence; tRNA-Phe gene, complete sequence; and D-loop, partial sequence; mitochondrial

CTTCTTGAACTAAATCAGTCATCGTAGCTTAATTCACAAAAGCATAACACTGAAAATGTTAATATGGACA

AAGAGTCCCGAATGACAAACAGACCAACCAAAAACCACGAGCACTATTACCTTCCCCCAAACAAAAAGAT

AATATGTACCACCCCCCAGCTATGTATTATAAGGCATTCATTTATCTGCCCCTAGCACCCATCCATTAGT

TCTTATTAGTCAGCATCTCACGTGAAATCACCATCCATTGTATCCATACTAACTATTACTAGTCTCAAGC

CCATACCTGGACACGGCTCACATTTATTGCTCTTCTTAGAGACCTCTGGTTATCACTCTCACGTACCCAT

CTTGCTATTGCCTGGACATTCTTTCCTCTTCTTAGAGGCCTCAACCCGCACCATATGGCTTCACTCATTC

ACGTCCGTAATCGCGGCATCCC

>EU034603.1 Crocodylus rhombifer strain LD150 tRNA-Pro gene, partial sequence; tRNA-Phe gene, complete sequence; and D-loop, partial sequence; mitochondrial

CTTCTTGAACTAAATCAGTCATCGTAGCTTAATTCACAAAAGCATAACACTGAAAATGTTAATATGGACA

AAGAGTCCCGAATGACAAACAGACCAACCAAAAACCACGAGCACTATTACCTTCCCCCAAACAAAAAGAT

AATATGTACCACCCCCCAGCTATGTATTATAAGGCATTCATTTATCTGCCCCTAGCACCCATCCATTAGT

TCTTATTAGTCAGCATCTCACGTGAAATCACCATCCATTGTATCCATACTAACTATTACTAGTCTCAAGC

CCATACCTGGACACGGCTCACATTTATTGCTCTTCTTAGAGACCTCTGGTTATCACTCTCACGTACCCAT

CTTGCTATTGCCTGGACATTCTTTCCTCTTCTTAGAGGCCTCAACCCGCACCATATGGCTTCACTCATTC

ACGTCCGTAATCGCGGCATCCC

>EU034602.1 Crocodylus rhombifer strain LD148 tRNA-Pro gene, partial sequence; tRNA-Phe gene, complete sequence; and D-loop, partial sequence; mitochondrial

CTTCTTGAACTAAATCAGTCATCGTAGCTTAATTCACAAAAGCATAACACTGAAAATGTTAATATGGACA

AAGAGTCCCGAATGACAAACAGACCAACCAAAAACCACGAGCACTATTACCTTCCCCCAAACAAAAAGAT

AATATGTACCACCCCCCAGCTATGTATTATAAGGCATTCATTTATCTGCCCCTAGCACCCATCCATTAGT

TCTTATTAGTCAGCATCTCACGTGAAATCACCATCCATTGTATCCATACTAACTATTACTAGTCTCAAGC

CCATACCTGGACACGGCTCACATTTATTGCTCTTCTTAGAGACCTCTGGTTATCACTCTCACGTACCCAT

CTTGCTATTGCCTGGACATTCTTTCCTCTTCTTAGAGGCCTCAACCCGCACCATATGGCTTCACTCATTC

ACGTCCGTAATCGCGGCATCCC

>EU034601.1 Crocodylus rhombifer strain LD147 tRNA-Pro gene, partial sequence; tRNA-Phe gene, complete sequence; and D-loop, partial sequence; mitochondrial

CTTCTTGAACTAAATCAGTCATCGTAGCTTAATTCACAAAAGCATAACACTGAAAATGTTAATATGGACA

AAGAGTCCCGAATGACAAACAGACCAACCAAAAACCACGAGCACTATTACCTTCCCCCAAACAAAAAGAT

AATATGTACCACCCCCCAGCTATGTATTATAAGGCATTCATTTATCTGCCCCTAGCACCCATCCATTAGT

TCTTATTAGTCAGCATCTCACGTGAAATCACCATCCATTGTATCCATACTAACTATTACTAGTCTCAAGC

CCATACCTGGACACGGCTCACATTTATTGCTCTTCTTAGAGACCTCTGGTTATCACTCTCACGTACCCAT

CTTGCTATTGCCTGGACATTCTTTCCTCTTCTTAGAGGCCTCAACCCGCACCATATGGCTTCACTCATTC

ACGTCCGTAATCGCGGCATCCC

>EU034600.1 Crocodylus rhombifer strain LD145 tRNA-Pro gene, partial sequence; tRNA-Phe gene, complete sequence; and D-loop, partial sequence; mitochondrial

CTTCTTGAACTAAATCAGTCATCGTAGCTTAATTCACAAAAGCATAACACTGAAAATGTTAATATGGACA

AAGAGTCCCGAATGACAAACAGACCAACCAAAAACCACGAGCACTATTACCTTCCCCCAAACAAAAAGAT

AATATGTACCACCCCCCAGCTATGTATTATAAGGCATTCATTTATCTGCCCCTAGCACCCATCCATTAGT

TCTTATTAGTCAGCATCTCACGTGAAATCACCATCCATTGTATCCATACTAACTATTACTAGTCTCAAGC

CCATACCTGGACACGGCTCACATTTATTGCTCTTCTTAGAGACCTCTGGTTATCACTCTCACGTACCCAT

CTTGCTATTGCCTGGACATTCTTTCCTCTTCTTAGAGGCCTCAACCCGCACCATATGGCTTCACTCATTC

ACGTCCGTAATCGCGGCATCCC

>EU034599.1 Crocodylus rhombifer strain LD125 tRNA-Pro gene, partial sequence; tRNA-Phe gene, complete sequence; and D-loop, partial sequence; mitochondrial

CTTCTTGAACTAAATCAGTCATCGTAGCTTAATTCACAAAAGCATAACACTGAAAATGTTAATATGGACA

AAGAGTCCCGAATGACAAACAGACCAACCAAAAACCACGAGCACTATTACCTTCCCCCAAACAAAAAGAT

AATATGTACCACCCCCCAGCTATGTATTATAAGGCATTCATTTATCTGCCCCTAGCACCCATCCATTAGT

TCTTATTAGTCAGCATCTCACGTGAAATCACCATCCATTGTATCCATACTAACTATTACTAGTCTCAAGC

CCATACCTGGACACGGCTCACATTTATTGCTCTTCTTAGAGACCTCTGGTTATCACTCTCACGTACCCAT

CTTGCTATTGCCTGGACATTCTTTCCTCTTCTTAGAGGCCTCAACCCGCACCATATGGCTTCACTCATTC

ACGTCCGTAATCGCGGCATCCC

>EU034598.1 Crocodylus rhombifer strain LD123 tRNA-Pro gene, partial sequence; tRNA-Phe gene, complete sequence; and D-loop, partial sequence; mitochondrial

CTTCTTGAACTAAATCAGTCATCGTAGCTTAATTCACAAAAGCATAACACTGAAAATGTTAATATGGACA

AAGAGTCCCGAATGACAAACAGACCAACCAAAAACCACGAGCACTATTACCTTCCCCCAAACAAAAAGAT

AATATGTACCACCCCCCAGCTATGTATTATAAGGCATTCATTTATCTGCCCCTAGCACCCATCCATTAGT

TCTTATTAGTCAGCATCTCACGTGAAATCACCATCCATTGTATCCATACTAACTATTACTAGTCTCAAGC

CCATACCTGGACACGGCTCACATTTATTGCTCTTCTTAGAGACCTCTGGTTATCACTCTCACGTACCCAT

CTTGCTATTGCCTGGACATTCTTTCCTCTTCTTAGAGGCCTCAACCCGCACCATATGGCTTCACTCATTC

ACGTCCGTAATCGCGGCATCCC

>EU034597.1 Crocodylus rhombifer strain LD100 tRNA-Pro gene, partial sequence; tRNA-Phe gene, complete sequence; and D-loop, partial sequence; mitochondrial

CTTCTTGAACTAAATCAGTCATCGTAGCTTAATTCACAAAAGCATAACACTGAAAATGTTAATATGGACA

AAGAGTCCCGAATGACAAACAGACCAACCAAAAACCACGAGCACTATTACCTTCCCCCAAACAAAAAGAT

AATATGTACCACCCCCCAGCTATGTATTATAAGGCATTCATTTATCTGCCCCTAGCACCCATCCATTAGT

TCTTATTAGTCAGCATCTCACGTGAAATCACCATCCATTGTATCCATACTAACTATTACTAGTCTCAAGC

CCATACCTGGACACGGCTCACATTTATTGCTCTTCTTAGAGACCTCTGGTTATCACTCTCACGTACCCAT

CTTGCTATTGCCTGGACATTCTTTCCTCTTCTTAGAGGCCTCAACCCGCACCATATGGCTTCACTCATTC

ACGTCCGTAATCGCGGCATCCC

>EU034596.1 Crocodylus rhombifer strain LD099 tRNA-Pro gene, partial sequence; tRNA-Phe gene, complete sequence; and D-loop, partial sequence; mitochondrial

CTTCTTGAACTAAATCAGTCATCGTAGCTTAATTCACAAAAGCATAACACTGAAAATGTTAATATGGACA

AAGAGTCCCGAATGACAAACAGACCAACCAAAAACCACGAGCACTATTACCTTCCCCCAAACAAAAAGAT

AATATGTACCACCCCCCAGCTATGTATTATAAGGCATTCATTTATCTGCCCCTAGCACCCATCCATTAGT

TCTTATTAGTCAGCATCTCACGTGAAATCACCATCCATTGTATCCATACTAACTATTACTAGTCTCAAGC

CCATACCTGGACACGGCTCACATTTATTGCTCTTCTTAGAGACCTCTGGTTATCACTCTCACGTACCCAT

CTTGCTATTGCCTGGACATTCTTTCCTCTTCTTAGAGGCCTCAACCCGCACCATATGGCTTCACTCATTC

ACGTCCGTAATCGCGGCATCCC

>EU034595.1 Crocodylus rhombifer strain LD069 tRNA-Pro gene, partial sequence; tRNA-Phe gene, complete sequence; and D-loop, partial sequence; mitochondrial

CTTCTTGAACTAAATCAGTCATCGTAGCTTAATTCACAAAAGCATAACACTGAAAATGTTAATATGGACA

AAGAGTCCCGAATGACAAACAGACCAACCAAAAACCACGAGCACTATTACCTTCCCCCAAACAAAAAGAT

AATATGTACCACCCCCCAGCTATGTATTATAAGGCATTCATTTATCTGCCCCTAGCACCCATCCATTAGT

TCTTATTAGTCAGCATCTCACGTGAAATCACCATCCATTGTATCCATACTAACTATTACTAGTCTCAAGC

CCATACCTGGACACGGCTCACATTTATTGCTCTTCTTAGAGACCTCTGGTTATCACTCTCACGTACCCAT

CTTGCTATTGCCTGGACATTCTTTCCTCTTCTTAGAGGCCTCAACCCGCACCATATGGCTTCACTCATTC

ACGTCCGTAATCGCGGCATCCC

>EU034594.1 Crocodylus rhombifer strain LD068 tRNA-Pro gene, partial sequence; tRNA-Phe gene, complete sequence; and D-loop, partial sequence; mitochondrial

CTTCTTGAACTAAATCAGTCATCGTAGCTTAATTCACAAAAGCATAACACTGAAAATGTTAATATGGACA

AAGAGTCCCGAATGACAAACAGACCAACCAAAAACCACGAGCACTATTACCTTCCCCCAAACAAAAAGAT

AATATGTACCACCCCCCAGCTATGTATTATAAGGCATTCATTTATCTGCCCCTAGCACCCATCCATTAGT

TCTTATTAGTCAGCATCTCACGTGAAATCACCATCCATTGTATCCATACTAACTATTACTAGTCTCAAGC

CCATACCTGGACACGGCTCACATTTATTGCTCTTCTTAGAGACCTCTGGTTATCACTCTCACGTACCCAT

CTTGCTATTGCCTGGACATTCTTTCCTCTTCTTAGAGGCCTCAACCCGCACCATATGGCTTCACTCATTC

ACGTCCGTAATCGCGGCATCCC

>EU034593.1 Crocodylus rhombifer strain LD042 tRNA-Pro gene, partial sequence; tRNA-Phe gene, complete sequence; and D-loop, partial sequence; mitochondrial

CTTCTTGAACTAAATCAGTCATCGTAGCTTAATTCACAAAAGCATAACACTGAAAATGTTAATATGGACA

AAGAGTCCCGAATGACAAACAGACCAACCAAAAACCACGAGCACTATTACCTTCCCCCAAACAAAAAGAT

AATATGTACCACCCCCCAGCTATGTATTATAAGGCATTCATTTATCTGCCCCTAGCACCCATCCATTAGT

TCTTATTAGTCAGCATCTCACGTGAAATCACCATCCATTGTATCCATACTAACTATTACTAGTCTCAAGC

CCATACCTGGACACGGCTCACATTTATTGCTCTTCTTAGAGACCTCTGGTTATCACTCTCACGTACCCAT

CTTGCTATTGCCTGGACATTCTTTCCTCTTCTTAGAGGCCTCAACCCGCACCATATGGCTTCACTCATTC

ACGTCCGTAATCGCGGCATCCC

>KM577702.1 Crocodylus rhombifer haplotype D D-loop, partial sequence; mitochondrial

CAACCAAAAACCACGAGCACTATTACCTTCCCCCAAACAAAAAGATAATATGTACCACCCCCCAGCTATG

TATTATAAGGCATTCATTTATCTGCCCCTAGCACCCATCCATTAGTTCTTACTAGTCAGCATCTCACGTG

AAATCACCATCCATTGTATCCATACTAACTATTACTAGTCTCAAGCCCATACCTGGACACGGCTCACATT

TATTGCTCTTCTTAGAGACCTCTGGTTATCACTCTCACGTACCCATCTTGCTATTGCCTGGACATTCTTT

CCTCTTCTTAGAGGCCTCAACCCGCACCATATGGCTTCACTCATTCACGTCCGTAATCGCGGCATCCCTT

TTCTTCAATGGCTATTGGTTCTTTATTTTTTTGGGGAGATCTCATCCACTACCCGGGGGCTTATATCTAA

AATCATTAAATCAAGGTGGTACATATTCCTTGCAGAAC

>JF315357.1 Crocodylus rhombifer isolate LSUMZ_H-21745 tRNA-Phe gene and D-loop, partial sequence; mitochondrial

GAGTCCCGAATGACAAACAGACCAACCAAAAACCACGAGCACTATTACCTTCCCCCAAACAAAAAGATAA

TATGTACCACCCCCCAGCTATGTATTATAAGGCATTCATTTATCTGCCCCTAGCACCCATCCATTAGTTC

TTATTAGTCAGCATCTCACGTGAAATCACCATCCATTGTATCCATACTAACTATTACTAGTCTCAAGCCC

ATACCTGGACACGGCTCACATTTATTGCTCTTCTTAGAGACCTCTGGTTATCACTCTCACGTACCCATCT

TGCTATTGCCTGGACATTCTTTCCTCTTCTTAGAGGCCTCAACCCGCACCATATGGCTTCACTCATTCAC

GTCCGTAATCGCGGCATCCCTTTTCTTCAATGGCTATTGGTTCTTTATTTTTTTGGGGAGATCTCATCCA

CTACCCGGGGGCTTATATCTAAAATCATTAAATCAAGGTGGTACATATTCCTTGCAGAACGGACCAGCTC

GGCTTTCATGTACATAAATATTTAATGCTCGTTATACATATTACTCTTTAATTAGGCCCCCCCTCCCCCC

AGTTTT

>JF315351.1 Crocodylus rhombifer isolate LSUMZ_H-21746 tRNA-Phe gene and D-loop, partial sequence; mitochondrial

GAGTCCCGAATGACAAACAGACCAACCAAAAACCACGAGCACTATTACCTTCCCCCAAACAAAAAGATAA

TATGTACCACCCCCCAGCTATGTATTATAAGGCATTCATTTATCTGCCCCTAGCACCCATCCATTAGTTC

TTATTAGTCAGCATCTCACGTGAAATCACCATCCATTGTATCCATACTAACTATTACTAGTCTCAAGCCC

ATACCTGGACACGGCTCACATTTATTGCTCTTCTTAGAGACCTCTGGTTATCACTCTCACGTACCCATCT

TGCTATTGCCTGGACATTCTTTCCTCTTCTTAGAGGCCTCAACCCGCACCATATGGCTTCACTCATTCAC

GTCCGTAATCGCGGCATCCCTTTTCTTCAATGGCTATTGGTTCTTTATTTTTTTGGGGAGATCTCATCCA

CTACCCGGGGGCTTATATCTAAAATCATTAAATCAAGGTGGTACATATTCCTTGCAGAACGGACCAGCTC

GGCTTTCATGTACATAAATATTTAATGCTCGTTATACATATTACTCTTTAATTAGGCCCCCCCTCCCCCC

AGTTTT

>JF315339.1 Crocodylus rhombifer isolate LSUMZ_H-21747 tRNA-Phe gene and D-loop, partial sequence; mitochondrial

GAGTCCCGAATGACAAACAGACCAACCAAAAACCACGAGCACTATTACCTTCCCCCAAACAAAAAGATAA

TATGTACCACCCCCCAGCTATGTATTATAAGGCATTCATTTATCTGCCCCTAGCACCCATCCATTAGTTC

TTATTAGTCAGCATCTCACGTGAAATCACCATCCATTGTATCCATACTAACTATTACTAGTCTCAAGCCC

ATACCTGGACACGGCTCACATTTATTGCTCTTCTTAGAGACCTCTGGTTATCACTCTCACGTACCCATCT

TGCTATTGCCTGGACATTCTTTCCTCTTCTTAGAGGCCTCAACCCGCACCATATGGCTTCACTCATTCAC

GTCCGTAATCGCGGCATCCCTTTTCTTCAATGGCTATTGGTTCTTTATTTTTTTGGGGAGATCTCATCCA

CTACCCGGGGGCTTATATCTAAAATCATTAAATCAAGGTGGTACATATTCCTTGCAGAACGGACCAGCTC

GGCTTTCATGTACATAAATATTTAATGCTCGTTATACATATTACTCTTTAATTAGGCCCCCCCTCCCCCC

AGTTTT

>EU499910.1 Crocodylus rhombifer isolate CrH tRNA-Pro and tRNA-Phe genes, complete sequence; and D-loop, partial sequence; mitochondrial

ACAACCGTTCAAGAAGGCAGACATAACTCTACTCTTCCGGCCCCCAAAGCCGACATTCTTATTAAACTAC

TTCTTGAACTAAATCAGTCATCGTAGCTTAATTCACAAAAGCATAACACTGAAAATGTTAATATGGACAA

AGAGTCCCGAATGACAAACAGACCAACCAAAAACCACGAGCACTATTACCTTCCCCCAAACAAAAAGATA

ATATGTACCACCCCCCAGCTATGTATTATAAGGCATTCATTTATCTGCCCCTAGCACCCATCCATTAGTT

CTTATTAGTCAGCATCTCACGTGAAATCACCATCCATTGTATCCATACTAACTATTACTAGTCTCAAGCC

CATACCTGGACACGGCTCACATTTATTGCTCTTCTTAGAGACCTCTGGTTATCACTCTCACGTACCCATC

TTGCTATTGCCTGGACATTCTTTCCTCTTCTTAGAGGCCTCAACCCGCACCATATGG

>EU034625.1 Crocodylus rhombifer x acutus strain RC051 tRNA-Pro gene, partial sequence; tRNA-Phe gene, complete sequence; and D-loop, partial sequence; mitochondrial

CTTCTTGAACTAAATCAGTCATCGTAGCTTAATTCACAAAAGCATAACACTGAAAATGTTAATATGGACA

AAGAGTCCCGAATGACAAACAGACCAACCAAAAACCACGAGCACTATTACCTTCCCCCAAACAAAAAGAT

AATATGTACCACCCCCCAGCTATGTATTATAAGGCATTCATTTATCTGCCCCTAGCACCCATCCATTAGT

TCTTATTAGTCAGCATCTCACGTGAAATCACCATCCATTGTATCCATACTAACTATTACTAGTCTCAAGC

CCATACCTGGACACGGCTCACATTTATTGCTCTTCTTAGAGACCTCTGGTTATCACTCTCACGTACCCAT

CTTGCTATTGCCTGGACATTCTTTCCTCTTCTTAGAGGCCTCAACCCGCACCATATGGCTTCACTCATTC

ACGTCCGTAATCGCGGCATCCC

>EU034624.1 Crocodylus rhombifer x acutus strain MF5267 tRNA-Pro gene, partial sequence; tRNA-Phe gene, complete sequence; and D-loop, partial sequence; mitochondrial

CTTCTTGAACTAAATCAGTCATCGTAGCTTAATTCACAAAAGCATAACACTGAAAATGTTAATATGGACA

AAGAGTCCCGAATGACAAACAGACCAACCAAAAACCACGAGCACTATTACCTTCCCCCAAACAAAAAGAT

AATATGTACCACCCCCCAGCTATGTATTATAAGGCATTCATTTATCTGCCCCTAGCACCCATCCATTAGT

TCTTATTAGTCAGCATCTCACGTGAAATCACCATCCATTGTATCCATACTAACTATTACTAGTCTCAAGC

CCATACCTGGACACGGCTCACATTTATTGCTCTTCTTAGAGACCTCTGGTTATCACTCTCACGTACCCAT

CTTGCTATTGCCTGGACATTCTTTCCTCTTCTTAGAGGCCTCAACCCGCACCATATGGCTTCACTCATTC

ACGTCCGTAATCGCGGCATCCC

>GU064610.1 Crocodylus rhombifer x acutus isolate MF5269 control region, partial sequence; mitochondrial

CAACCAAAAACCACGAGCACTATTACCTTCCCCCAAACAAAAAGATAATATGTACCACCCCCCAGCTATG

TATTATAAGGCATTCATTTATCTGCCCCTAGCACCCATCCATTAGTTCTTATTAGTCAGCATCTCACGTG

AAATCACCATCCATTGTATCCATACTAACTATTACTAGTCTCAAGCCCATACCTGGACACGGCTCACATT

TATTGCTCTTCTTAGAGACCTCTGGTTATCACTCTCACGTACCCATCTTGCTATTGCCTGGACATTCTTT

CCTCTTCTTAGAGGCCTCAACCCGCACCATATGGCTTCACTCATTCACGTCCGTAATCGCGGCATCCCTT

TTCTTCAATGGCTATTGGTTCTTTATTTTTTTGGGGAGATCTCATCCACTACCCGGGGGCTTATATCTAA

AATCATTAAATCAAGGTGGTACATATTCCTTGCAGAAC

>GU064609.1 Crocodylus rhombifer x acutus isolate MF5268 control region, partial sequence; mitochondrial

CAACCAAAAACCACGAGCACTATTACCTTCCCCCAAACAAAAAGATAATATGTACCACCCCCCAGCTATG

TATTATAAGGCATTCATTTATCTGCCCCTAGCACCCATCCATTAGTTCTTATTAGTCAGCATCTCACGTG

AAATCACCATCCATTGTATCCATACTAACTATTACTAGTCTCAAGCCCATACCTGGACACGGCTCACATT

TATTGCTCTTCTTAGAGACCTCTGGTTATCACTCTCACGTACCCATCTTGCTATTGCCTGGACATTCTTT

CCTCTTCTTAGAGGCCTCAACCCGCACCATATGGCTTCACTCATTCACGTCCGTAATCGCGGCATCCCTT

TTCTTCAATGGCTATTGGTTCTTTATTTTTTTGGGGAGATCTCATCCACTACCCGGGGGCTTATATCTAA

AATCATTAAATCAAGGTGGTACATATTCCTTGCAGAAC

>GU064608.1 Crocodylus rhombifer x acutus isolate MF5266 control region, partial sequence; mitochondrial

CAACCAAAAACCACGAGCACTATTACCTTCCCCCAAACAAAAAGATAATATGTACCACCCCCCAGCTATG

TATTATAAGGCATTCATTTATCTGCCCCTAGCACCCATCCATTAGTTCTTATTAGTCAGCATCTCACGTG

AAATCACCATCCATTGTATCCATACTAACTATTACTAGTCTCAAGCCCATACCTGGACACGGCTCACATT

TATTGCTCTTCTTAGAGACCTCTGGTTATCACTCTCACGTACCCATCTTGCTATTGCCTGGACATTCTTT

CCTCTTCTTAGAGGCCTCAACCCGCACCATATGGCTTCACTCATTCACGTCCGTAATCGCGGCATCCCTT

TTCTTCAATGGCTATTGGTTCTTTATTTTTTTGGGGAGATCTCATCCACTACCCGGGGGCTTATATCTAA

AATCATTAAATCAAGGTGGTACATATTCCTTGCAGAAC

>EU034586.1 Crocodylus cf. rhombifer strain MF347 tRNA-Pro gene, partial sequence; tRNA-Phe gene, complete sequence; and D-loop, partial sequence; mitochondrial

CTTCTTGAACTAAATCAGTCATCGTAGCTTAATTCACAAAAGCATAACACTGAAAATGTTAATATGGACA

AAGAGTCCCGAATGACAAACAGACCAGCCAAAAACCACGAACACTATTACCTTTCCCCAAACAAAAAGAT

AATATGTACCACCCCCCAGCTATGTATTATAAGGCATTCATTTATCTGCCCCTAGCACCCATCCATTAGT

TCTTATTAGTCAGCATCTCACGTGAAATCACCATCCATTGTATCCATACTAACTATTACTAGTCTCAAGC

CCATACCTGGACACGGCTCACATTCATTGCTCTTCTTGGAGACCTCTGGTTATCACTCTCACGTACCCAT

CTTGCTATTGCCTGGACATTCTTTCCTCTTCTTAGAGGCCTCAACCCGCACCATATGACTTCACTCATTT

ACGTCCGTAATCGCGGCATCCC

>EU034587.1 Crocodylus cf. rhombifer strain MF348 tRNA-Pro gene, partial sequence; tRNA-Phe gene, complete sequence; and D-loop, partial sequence; mitochondrial

CTTCTTGAACTAAATCAGTCATCGTAGCTTAATTCACAAAAGCATAACACTGAAAATGTTAATATGGACA

AAGAGTCCCGAATGACAAACAGACCAGCCAAAAACCACGAACACTATTACCTTTCCCCAAACAAAAAGAT

AATATGTACCACCCCCCAGCTATGTATTATAAGGCATTCATTTATCTGCCCCTAGCACCCATCCATTAGT

TCTTATTAGTCAGCATCTCACGTGAAATCACCATCCATTGTATCCATACTAACTATTACTAGTCTCAAGC

CCATACCTGGACACGGCTCACATTCATTGCTCTTCTTGGAGACCTCTGGTTATCACTCTCACGTACCCAT

CTTGCTATTGCCTGGACATTCTTTCCTCTTCTTAGAGGCCTCAACCCGCACCATATGACTTCACTCATTT

ACGTCCGTAATCGCGGCATCCC

>EU034588.1 Crocodylus cf. rhombifer strain SP014 tRNA-Pro gene, partial sequence; tRNA-Phe gene, complete sequence; and D-loop, partial sequence; mitochondrial

CTTCTTGAACTAAATCAGTCATCGTAGCTTAATTCACAAAAGCATAACACTGAAAATGTTAATATGGACA

AAGAGTCCCGAATGACAAACAGACCAGCCAAAAACCACGAACACTATTACCTTTCCCCAAACAAAAAGAT

AATATGTACCACCCCCCAGCTATGTATTATAAGGCATTCATTTATCTGCCCCTAGCACCCATCCATTAGT

TCTTATTAGTCAGCATCTCACGTGAAATCACCATCCATTGTATCCATACTAACTATTACTAGTCTCAAGC

CCATACCTGGACACGGCTCACATTCATTGCTCTTCTTGGAGACCTCTGGTTATCACTCTCACGTACCCAT

CTTGCTATTGCCTGGACATTCTTTCCTCTTCTTAGAGGCCTCAACCCGCACCATATGACTTCACTCATTT

ACGTCCGTAATCGCGGCATCCC

>EU034589.1 Crocodylus cf. rhombifer strain PM008 tRNA-Pro gene, partial sequence; tRNA-Phe gene, complete sequence; and D-loop, partial sequence; mitochondrial

CTTCTTGAACTAAATCAGTCATCGTAGCTTAATTCACAAAAGCATAACACTGAAAATGTTAATATGGACA

AAGAGTCCCGAATGACAAACAGACCAGCCAAAAACCACGAACACTATTACCTTTCCCCAAACAAAAAGAT

AATATGTACCACCCCCCAGCTATGTATTATAAGGCATTCATTTATCTGCCCCTAGCACCCATCCATTAGT

TCTTATTAGTCAGCATCTCACGTGAAATCACCATCCATTGTATCCATACTAACTATTACTAGTCTCAAGC

CCATACCTGGACACGGCTCACATTCATTGCTCTTCTTGGAGACCTCTGGTTATCACTCTCACGTACCCAT

CTTGCTATTGCCTGGACATTCTTTCCTCTTCTTAGAGGCCTCAACCCGCACCATATGACTTCACTCATTT

ACGTCCGTAATCGCGGCATCCC

>EU034590.1 Crocodylus cf. rhombifer strain PM031 tRNA-Pro gene, partial sequence; tRNA-Phe gene, complete sequence; and D-loop, partial sequence; mitochondrial

CTTCTTGAACTAAATCAGTCATCGTAGCTTAATTCACAAAAGCATAACACTGAAAATGTTAATATGGACA

AAGAGTCCCGAATGACAAACAGACCAGCCAAAAACCACGAACACTATTACCTTTCCCCAAACAAAAAGAT

AATATGTACCACCCCCCAGCTATGTATTATAAGGCATTCATTTATCTGCCCCTAGCACCCATCCATTAGT

TCTTATTAGTCAGCATCTCACGTGAAATCACCATCCATTGTATCCATACTAACTATTACTAGTCTCAAGC

CCATACCTGGACACGGCTCACATTCATTGCTCTTCTTGGAGACCTCTGGTTATCACTCTCACGTACCCAT

CTTGCTATTGCCTGGACATTCTTTCCTCTTCTTAGAGGCCTCAACCCGCACCATATGACTTCACTCATTT

ACGTCCGTAATCGCGGCATCCC

>EU034591.1 Crocodylus cf. rhombifer strain PM037 tRNA-Pro gene, partial sequence; tRNA-Phe gene, complete sequence; and D-loop, partial sequence; mitochondrial

CTTCTTGAACTAAATCAGTCATCGTAGCTTAATTCACAAAAGCATAACACTGAAAATGTTAATATGGACA

AAGAGTCCCGAATGACAAACAGACCAGCCAAAAACCACGAACACTATTACCTTTCCCCAAACAAAAAGAT

AATATGTACCACCCCCCAGCTATGTATTATAAGGCATTCATTTATCTGCCCCTAGCACCCATCCATTAGT

TCTTATTAGTCAGCATCTCACGTGAAATCACCATCCATTGTATCCATACTAACTATTACTAGTCTCAAGC

CCATACCTGGACACGGCTCACATTCATTGCTCTTCTTGGAGACCTCTGGTTATCACTCTCACGTACCCAT

CTTGCTATTGCCTGGACATTCTTTCCTCTTCTTAGAGGCCTCAACCCGCACCATATGACTTCACTCATTT

ACGTCCGTAATCGCGGCATCCC

>EU034592.1 Crocodylus cf. rhombifer strain GC001 tRNA-Pro gene, partial sequence; tRNA-Phe gene, complete sequence; and D-loop, partial sequence; mitochondrial

CTTCTTGAACTAAATCAGTCATCGTAGCTTAATTCACAAAAGCATAACACTGAAAATGTTAATATGGACA

AAGAGTCCCGAATGACAAACAGACCAGCCAAAAACCACGAACACTATTACCTTTCCCCAAACAAAAAGAT

AATATGTACCACCCCCCAGCTATGTATTATAAGGCATTCATTTATCTGCCCCTAGCACCCATCCATTAGT

TCTTATTAGTCAGCATCTCACGTGAAATCACCATCCATTGTATCCATACTAACTATTACTAGTCTCAAGC

CCATACCTGGACACGGCTCACATTCATTGCTCTTCTTGGAGACCTCTGGTTATCACTCTCACGTACCCAT

CTTGCTATTGCCTGGACATTCTTTCCTCTTCTTAGAGGCCTCAACCCGCACCATATGACTTCACTCATTT

ACGTCCGTAATCGCGGCATCCC

>EU034626.1 Crocodylus acutus strain RC023 tRNA-Pro gene, partial sequence; tRNA-Phe gene, complete sequence; and D-loop, partial sequence; mitochondrial

CTTCTTGAACTAACTCAGTCATCGTAGCTTAACTCACAAAAGCATAACACTGAAAATGTTAATATGGACA

AGGAGTCCCGAATGACAAACGGACTAACCAAAAACTACGAGCACTATTACCTTTCCCCAGACAAAAAGAT

AATATGTACCACCCCCCAGCTATGTATTATAAGGCATTCATTTATTTGCCCCTAACACCCATCCATTAGT

TCTTATTAATCAGCATCTCACGTGAAATCACCATCCATTGTATCCATACTAACTATTACTAGTCTCAAGC

CCATACCTGGACACGACTCACATTCATTGCTCTTCTTAGAGACCTCTGGTTATCACTCTCACGTACCCAT

CTTGCTATTGCCTGGACATTCTTTCCTCTTCTTAGAGGCCTCAACCCGCACCATATGGTTTCACTCATTC

ACGTCCGTGATCGCGGCATCCC

>EU034627.1 Crocodylus acutus strain RC210 tRNA-Pro gene, partial sequence; tRNA-Phe gene, complete sequence; and D-loop, partial sequence; mitochondrial

CTTCTTGAACTAAGTCAGTCATCGTAGCTTAACTCACAAAAGCATAACACTGAAAATGTTAATATGGACA

AGGAGTCCCGAATGACAAACGGACTAACCAAAAACTACGAGCACTATTACCTTTCCCCAGACAAAAAGAT

AATATGTACCACCCCCCAGCTATGTATTATAAGGCATTCATTTATTTGCCCCTAACACCCATCCATTAGT

TCTTATTAATCAGCATCTCACGTGAAATCACCATCCATTGTATCCATACTAACTATTACTAGTCTCAAGC

CCATACCTGGACACGACTCACATTCATTGCTCTTCTTAGAGACCTCTGGTTATCACTCTCACGTACCCAT

CTTGCTATTGCCTGGACATTCTTTCCTCTTCTTAGAGGCCTCAACCCGCACCATATGGTTTCACTCATTC

ACGTCCGTGATCGCGGCATCCC
