# Supplementary material for: Environmental DNA-based biomonitoring of Cuban Crocodylus and their accompanying vertebrate fauna from Zapata Swamp, Cuba
Source: Sci Rep. 2023 Nov 22;13:20438. doi: 10.1038/s41598-023-47675-8 (PMC10665403; doi:10.1038/s41598-023-47675-8)
Supplement: Supplementary file 2 — Supplementary Information 2. [file 41598_2023_47675_MOESM2_ESM.docx]

Supplementary Information 2. Analytical parameters (trimming, filtering, paired-end and other parameters) configured for metabarcoding data analysis in mBRAVE when using COI markers.

| Analytical  parameters | Specific settings | Values |
| --- | --- | --- |
| Trimming | Trim Front | 25 bp |
|  | Trim End | 26 bp |
|  | Trim Length | 450 bp |
|  | Primer Masking | Off |
| Filtering | Min QV | 20 qv |
|  | Min Length | 100 bp |
|  | Max Bases with Low QV(<20) | 20% |
|  | Max Bases with Ultra Low QV(10) | 5 % |
| Other Parameters | Pre-Clustering Threshold | Off |
|  | ID Distance Threshold | 3 % |
|  | Exclude from OTU Threshold | 3 % |
|  | Minimum OTU Size | 5 |
|  | OTU Threshold | 2 % |
| Paired-End | Paired End Merging | Merge |
|  | Assembler Min Overlap | 5 bp |
|  | Assembler Max Substitution | 5 bp |
